# CCL3 secreted by hepatocytes promotes the metastasis of intrahepatic cholangiocarcinoma by VIRMA-mediated N6-methyladenosine (m^6^A) modification

**DOI:** 10.1186/s12967-023-03897-y

**Published:** 2023-01-23

**Authors:** Shurui Zhou, Kege Yang, Shaojie Chen, Guoda Lian, Yuzhou Huang, Hanming Yao, Yue Zhao, Kaihong Huang, Dong Yin, Haoming Lin, Yaqing Li

**Affiliations:** 1 Guangdong Provincial Key Laboratory of Malignant Tumor Epigenetics and Gene Regulation, Sun Yat-Sen Memorial Hospital, Sun Yat-Sen University, Guangzhou, 510120 China; 2 Department of Gastroenterology, Sun Yat-Sen Memorial Hospital, Sun Yat-Sen University, Guangzhou, 510120 China; 3 Present Address: Medical Research Center, Sun Yat-Sen Memorial Hospital, Sun Yat-Sen University, Guangzhou, 510120 China; 4 Present Address: Department of Pancreato-Biliary Surgery, Sun Yat-Sen Memorial Hospital, Sun Yat-Sen University, Guangzhou, 510120 China

**Keywords:** Tumor microenvironment, Intrahepatic cholangiocarcinoma, m^6^A methylation, Vir-like m^6^A methyltransferase associated, Metastasis

## Abstract

**Background:**

Intrahepatic cholangiocarcinoma (ICC) is a malignant disease characterized by onset occult, rapid progression, high relapse rate, and high mortality. However, data on how the tumor microenvironment (TME) regulates ICC metastasis at the transcriptomic level remains unclear. This study aimed to explore the mechanisms and interactions between hepatocytes and ICC cells.

**Methods:**

We analyzed the interplay between ICC and liver microenvironment through cytokine antibody array analysis. Then we investigated the role of N6-methyladenosine (m^6^A) modification and the downstream target in vitro, in vivo experiments, and in clinical specimens.

**Results:**

Our study demonstrated that cytokine CCL3, which is secreted by hepatocytes, promotes tumor metastasis by regulating m^6^A modification via vir-like m^6^A methyltransferase associated (VIRMA) in ICC cells. Moreover, immunohistochemical analyses showed that VIRMA correlated with poor outcomes in ICC patients. Finally, we confirmed both in vitro and in vivo that CCL3 could activate VIRMA and its critical downstream target SIRT1, which fuels tumor metastasis in ICC.

**Conclusions:**

In conclusion, our results enhanced our understanding of the interaction between hepatocytes and ICC cells, and revealed the molecular mechanism of the CCL3/VIRMA/SIRT1 pathway via m^6^A-mediated regulation in ICC metastasis. These studies highlight potential targets for the diagnosis, treatment, and prognosis of ICC.

**Supplementary Information:**

The online version contains supplementary material available at 10.1186/s12967-023-03897-y.

## Introduction

Intrahepatic cholangiocarcinoma (ICC) emanates from the epithelial lining of the intrahepatic biliary tree, and is a malignant disease characterized by onset occult, rapid progression, high relapse rate, and high mortality [1, 2]. As the second most common primary liver cancer, ICC accounts for about 3% of all gastrointestinal malignancies, and recent years have seen a rapid increase in ICC-related morbidity and mortality [3, 4]. Thus, there is an urgent need to explore the molecular mechanisms mediating the development of ICC, which will lay the basis for identifying novel diagnostic biomarkers and validating therapeutic targets.

Tumor metastasis is one of the most important indicators of tumor progression and a leading cause of mortality in cancer patients. Besides, the tumor microenvironment (TME) has been shown to play a key role in driving tumor development and metastasis [5]. ICC is more prone to intrahepatic spreading and metastasis as compared with extrahepatic cholangiocarcinoma. The ICC cells break through the wall of the intrahepatic bile duct and invade the liver parenchyma. The ICC liver microenvironment is mainly composed of liver parenchymal cells, which account for 85%, and non-parenchymal cells [6]. Previous data have demonstrated that liver cells can modulate the TME via the secretion of cytokines, chemokines, and other tumor-regulating factors which are vital for tumor metastasis [7–9]. Other studies have shown that various cytokines and chemokines such as CCL2 and CXCL12 in the liver microenvironment mediate many physiological and pathological processes in cholangiocarcinoma [10, 11].

Chemokines, a family of small proteins (8–10 kDa) that interact with specific G-protein–coupled chemokine receptors, are key components of the TME [12]. The chemokines play an important role in processes controlled at epigenetic, transcriptional, and post-transcriptional regulation levels, which affect the occurrence and development of tumor cells [13]. In addition, there has been an association between extracellular signals and N6-methyladenosine (m^6^A) modification [14, 15]. For example, TGF-β can directly modulate the m^6^A dynamics which trigger cancer proliferation and metastasis [16, 17].

The m^6^A methylation occurs on the sixth N of adenylate (A) in RNA, which is the most universal and abundant epigenetic modification in eukaryotic mRNA [18, 19]. The m^6^A RNA methylation is a dynamic and reversible process mainly mediated by the methyltransferase complex. The process is mediated by m^6^A demethylases and is related to diverse cellular functions by recognizing and binding the m^6^A reader proteins [20]. The m^6^A methyltransferase complex consists of METTL3, METTL14, VIRMA (vir-like m^6^A methyltransferase associated), and WTAP, while the m^6^A demethylases comprise FTO and AlkBH5. m^6^A methyltransferase includes YTH domain-containing family proteins (YTHDFs, comprising YTHDF1/2/3) and insulin-like growth factor 2 binding proteins (IGF2BPs, comprising IGF2BP1/2/3), which can specifically recognize m^6^A modification sites and recruit related proteins to regulate the stability of mRNA, nuclear output or translation efficiency. Another study suggested that the m^6^A modification and m^6^A binding proteins participate in a range of physiological and pathological processes such as DNA damage [21], embryonic development [22], immune response [23], tumorigenesis, or tumor progression [24, 25]. Besides, m^6^A methylation plays an essential role in the promotion and metastasis of many types of malignancies such as gastric cancer [26], hepatocellular cancer [27], ovarian cancer [28], breast cancer [29], and colorectal cancer [30, 31], thus is a potential target for cancer therapy. For instance, VIRMA promotes tumor development in breast cancer [32], while METTL3-mediated m^6^A modification of HDGF mRNA modulates the progression of gastric cancer [26]. To date, however, data on the biological roles and mechanisms of m^6^A modification in ICC remain unclear.

In this study, we analyzed the interplay between ICC and liver microenvironment and investigated the role of m^6^A modification in ICC. Our study demonstrated that CCL3, which is secreted by hepatocytes, promotes tumor metastasis by fueling m^6^A modification in ICC. Moreover, we showed that VIRMA was a major modulator in the m^6^A modification, which correlated with poor outcomes in ICC patients and promoted ICC metastasis. In addition, SIRT1 was shown to be a critical downstream target of VIRMA, which fuels tumor metastasis. Taken together, our data enhanced our understanding of the interaction between hepatocytes and ICC cells, and uncovered the molecular mechanism of VIRMA-mediated m^6^A modification in ICC metastasis.

## Materials and methods

### Tissue specimens and clinical data

Ethical approval for this study was provided by the Institutional Review Board of Sun Yat-sen Memorial Hospital. A total of 110 paraffin-embedded ICC specimens and corresponding adjacent normal tissues were obtained from Sun Yat-sen Memorial Hospital, Sun Yat-sen University between January 2011 and December 2020.

### Cell culture

Immortalized normal liver epithelial cells (THLE3) and human normal bile duct epithelial cell lines (HIBEpiC) were obtained from the American Type Culture Collection (ATCC, Manassas, VA, USA). The cells were cultured as specified by respective manufacturers. Two intrahepatic cholangiocarcinoma cell lines (HuCCT1 and RBE) were acquired from the Cell Bank of the Chinese Academy of Sciences, Shanghai, China. The cell lines were grown at 37 °C under 5% CO_2_ in a 1640 medium supplemented with 10% fetal bovine serum (FBS, Hyclone, USA). The cells were seeded at a density of 5 × 10^5^ cells in a T25 culture flask and passaged every 4–5 days.

### Transwell assays

Here, we employed both the transwell migration and invasion assays. The ICC cells were inoculated in the upper transwell chamber (Corning, USA) with an indicated density of 3–5 × 10^4^ cells per well in the 24-well plates, with or without Matrigel (BD Biosciences, USA), and incubated for 24 h. Thereafter, unmigrated and uninvaded ICC cells were removed with cotton swabs. The ICC cells on the bottom surface were then fixed in methanol for 10 min, at room temperature, followed by staining with 0.5% crystal violet. The experiments were carried out three times and in triplicate.

### Quantitative real-time PCR

Total RNA was extracted from tissues and cells using TRIzol Reagent (Invitrogen, USA), following the manufacturer’s instructions. Quantitative real-time PCR (qRT-PCR) was performed using PrimeScript RT reagent Kit and SYBR Premix Ex Taq (Takara, Japan), and employed primer sequences as shown in Additional file 1: Table S1. Results were normalized to GAPDH expression and calculated using the 2^–ΔΔCt^ method. The experiments were repeated at least three times.

### Plasmid construction

Full-length VIRMA (Ensembl: ENSG00000164944) cDNA was designed and synthesized by GenePharma (Shanghai, China), and then the cloned fragments were ligated into pcDNA3.1. Lipofectamine 3000 (Invitrogen, USA) was served as the role of transfecting the plasmids into the ICC cells, following the manufacturer’s protocol. Small hairpin RNA (shRNA) of VIRMA (shVIRMA) and negative control (shNC) were designed as shown in Additional file 2: Table S2 and synthesized by GenePharma (Shanghai, China). In order to acquire stable VIRMA-knockdown ICC cell lines, the cells were infected with lentiviral particles and then selected under 8 μg/ml puromycin pressure.

### Animal experiments

All animal experiments were approved by the Animal Research Committee of Sun Yat-sen University Cancer Center. Female Balb/c nude mice (Balb/c-nu, weighing ~ 15–20 g), were purchased from the Guangdong Medical Laboratory Animal Center (Guangzhou, China). To generate a subcutaneous xenograft tumor model, we injected 5 × 10^6^ transfected HuCCT1 cells, which were suspended in 0.2 ml of PBS, into the flank of nude mice (five mice per group). On the other hand, an orthotopic xenograft tumor model was established by inoculating 3 × 10^6^ transfected HuCCT1 cells in 50 μl of PBS into the liver of nude mice (five mice per group). For the treated groups, HuCCT1 cells were treated with CCL3 (100 ng/ml) for 6 h before inoculation. For subcutaneous injection, the intratumoral multiple-point injection of CCL3 (20 mg/kg) diluted in 50 μl PBS was performed every 5 days. The control groups were treated with PBS. Subcutaneous tumor size was measured twice a week. After in vivo fluorescence imaging, all the study mice were sacrificed with the in vivo imaging system (IVIS) spectrum after four weeks. The mice tumors and organs were dissected, photographed, weighed, and stained.

Additional methods and experimental details are described in Additional file 4.

## Results

### Adjacent-cancer hepatocytes promote malignant progression of ICC

**Fig. 1:**
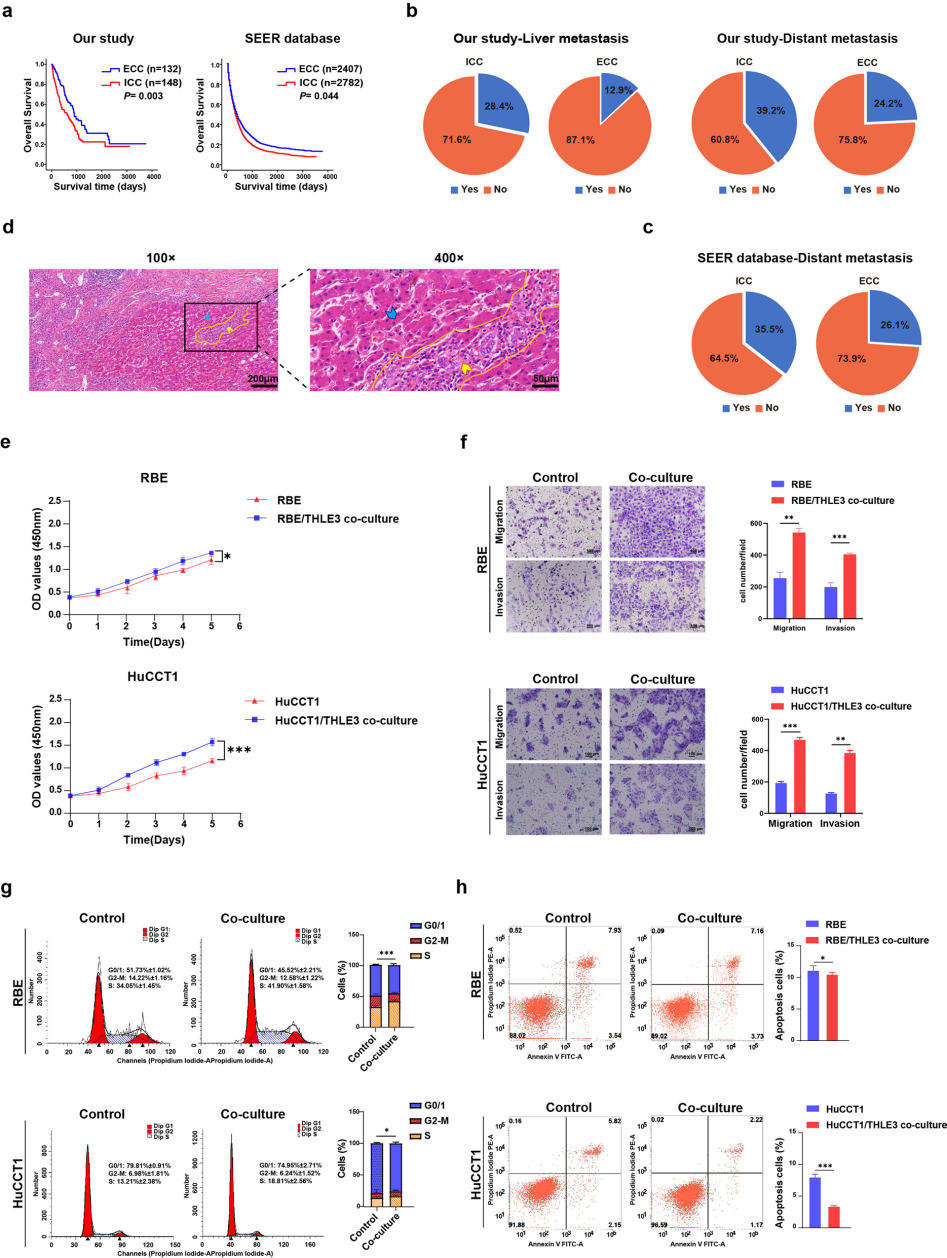
Adjacent-cancer hepatocytes promote the malignant progression of ICC. **a** Kaplan–Meier survival curves of cholangiocarcinoma patients in our hospital and SEER database. **b** Comparison of the cumulative percentage of patients who developed liver metastasis (left) or distant metastasis (right) between patients with ICC and ECC in our hospital. **c** Comparison of the cumulative percentage of patients who developed distant metastasis between patients with ICC and ECC in the SEER database. **d** Representative images of H&E-stained ICC tissue sections. Yellow arrowheads reveal infiltration of ICC cells within adjacent-cancer hepatocytes (blue arrows). Scale bars, 200 μm (left) and 50 μm (right). Human ICC cell line RBE and HuCCT1 were separately co-cultured with THLE3 hepatocytes. **e** CCK8 assays were performed to assess cell viability. **f** Transwell assays were employed to detect migratory and invasive abilities. Representative images (left) and quantification (right) of Transwell assays are shown. Scale bars, 100 μm. **g** Cell cycle progression and **h** cell apoptosis were measured using flow cytometry. Representative photographs (left) and quantification (right) are shown. ICC, intrahepatic cholangiocarcinoma; ECC, extrahepatic cholangiocarcinoma. *P < 0.05 **P < 0.01, ***P < 0.001

This study analyzed a total of 280 cholangiocarcinoma patient records with complete follow-up and clinical data from Sun Yat-sen Memorial Hospital of Sun Yat-sen University between 2009 and 2018 (Fig. 1). The data demonstrated that patients with ICC had a worse prognosis compared to those with ECC (Fig. 1a). In addition, compared to the ECC patients, patients with ICC had a higher chance of distant metastasis (ICC vs ECC: 39.2% vs 24.2%) and liver metastases (ICC vs ECC: 28.4% vs 12.9%) (Fig. 1b). Analysis of the SEER database showed the same trend in survival or prognosis of ICC and ECC patients. The SEER database analysis demonstrated that the median survival of the ICC patients was significantly shorter than that of the patients with ECC (Fig. 1a). Besides, ICC patients were significantly more susceptible to distant metastasis (ICC vs ECC: 35.5% vs 26.1%) (Fig. 1c). These findings showed that ICC is more prone to metastasis and has a worse prognosis than ECC. Thus, we hypothesized that these differences in disease development might be due to differential TME. Analysis of clinical data showed that hepatocytes, an important component of the liver microenvironment, played a crucial role in ICC metastasis. As shown in Fig. 1d, our data demonstrated that the ICC cells grew infiltratively along the intrahepatic bile duct, and then into adjacent liver parenchyma, which was in contact with hepatocytes.

To dissect the functional relationship between hepatocytes and ICC development and progression, we co-cultured the ICC cell lines RBE and HuCCT1 with hepatocytes (THLE3). The growth of the RBE and HuCCT1 cells after co-culture with THLE3 was measured using cell counting kit-8 (CCK-8) assays. The data showed that the co-culture with THLE3 significantly promoted the proliferation of RBE and HuCCT1 cells (Fig. 1e). Moreover, the transwell assay revealed that co-culture with THLE3 robustly enhanced the migratory and invasive ability of the ICC cells (Fig. 1f). In addition, we conducted cell cycle progression and cell apoptosis analysis by flow cytometry. As indicated in Fig. 1g, the co-culture with THLE3 resulted in a significant increase in S-phase cells and a reduction in cells at G0/1 phases. The apoptosis assays showed that the percentage of apoptotic cells was significantly higher in the co-culture group (Fig. 1h). Overall, these data indicated that hepatocytes promote the metastasis of ICC.

### Hepatocyte-secreted CCL3 promotes migration and invasion of the ICC cells

**Fig. 2:**
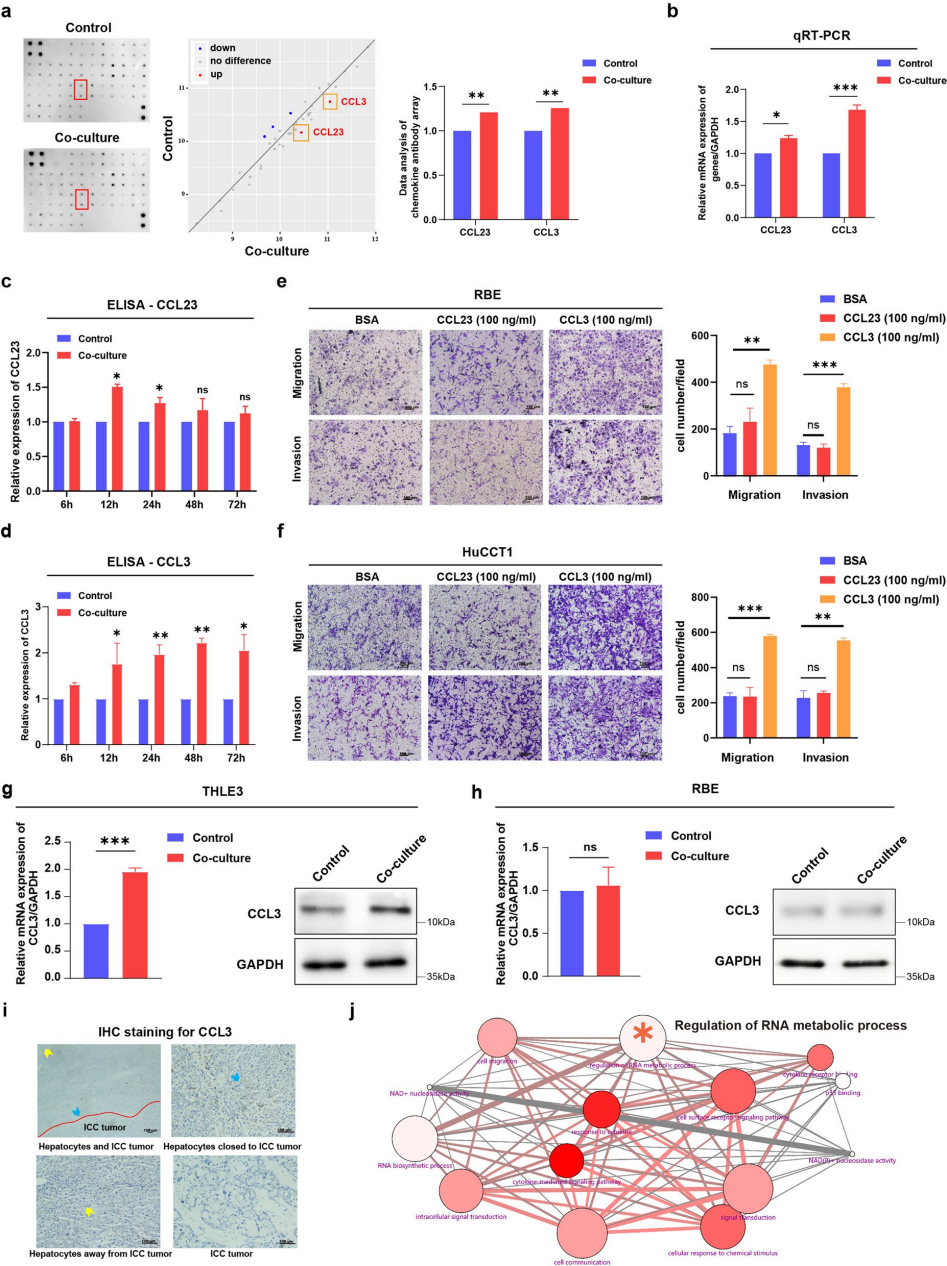
CCL3 secreted by adjacent-cancer hepatocytes promotes ICC migration and invasion. **a** After co-culture with ICC cells, conditioned supernatants secreted by hepatocytes were analyzed for cytokine levels through enzyme-linked immunosorbent assay (ELISA)-based cytokine antibody arrays (Raybiotech). Scan of original blots (left), data analysis schematic (middle), and representative differentially expressed proteins (right) of cytokine antibody arrays (Additional file 3: Fig. S1). **b** Measurements of levels of cytokine mRNA in THLE3 cells after co-culture with ICC cells were assayed by qRT-PCR. **c**, **d** Supernatants were collected and the cytokine secretion level of **c** CCL23 and **d** CCL3 were measured in RBE cells through ELISA. **e**, **f** Transwell migration assay and matrigel invasion assay **e** RBE cells and **f** HuCCT1 cells stimulated with CCL23 (100 ng/ml) or CCL3 (100 ng/ml). Representative images (left) and quantification (right) of Transwell assays are shown. Scale bars, 100 μm. **g** qRT-PCR and western blot analysis of CCL3 expression in THLE3 hepatocytes following co-culture with ICC cells. **h** qRT-PCR and western blot analysis of CCL3 expression in RBE cells following the co-culture. **i** Representative images of IHC staining for CCL3 protein of ICC tumor tissues and their adjacent liver tissues. Red curve: intrahepatic cholangiocarcinoma. Blue arrow, adjacent-cancer hepatocytes; yellow arrow, distant hepatocytes. Scale bars: 100 μm. **j** Gene ontology (GO) enrichment analysis was performed on the genes that interacted with CCL3. RBE, HuCCT1, human ICC cell lines; THLE3, human hepatocytes cell line. *P < 0.05 **P < 0.01, ***P < 0.001.

To assess the key chemotactic molecule in hepatocytes that affect the metastasis of ICC cells, we employed the cytokine antibody assay to examine the level of cytokines secreted by hepatocytes after co-culture with the ICC cells. As shown in Fig. 2a and Additional file 3: Fig. S1, our results showed a high secretion of cytokine CCL3 and CCL23 in hepatocytes after co-culture with RBE, an ICC cell. We then used qRT-PCR and ELISA to verify the prominent hypersecretion of cytokine CCL3 in supernatants collected from the THLE3 cell line after co-culture with the ICC cells (Fig. 2b and d), while a slightly elevated level of CCL23 secretion was observed in Fig. 2c. Next, to evaluate the role of different cytokines in the regulation of migration and invasion ability of the ICC cells, we conducted transwell migration assays and matrigel invasion assays of the RBE cells and HuCCT1 cells stimulated with CCL3 and CCL23. Our findings showed that CCL3 significantly enhanced the migration and invasion ability of HuCCT1 and RBE cells compared with the control group (Fig. 2e–f). On the contrary, there was no significant difference between the control and the treatment group with CCL23, which indicated that CCL3 was a critical factor in ICC infiltration and metastasis.

The expression of CCL3 in the hepatocytes was significantly upregulated after co-culture with the ICC cells, while there was no significant correlation in the CCL3 expression in ICC cells after co-culture with THLE3 (Fig. 2g, h). Moreover, immunohistochemical staining was performed to profile the localization of CCL3 protein in ICC tumor and adjacent liver tissues. The data showed that the CCL3 was mainly expressed in the cytoplasm of hepatocytes and intercellular matrix adjacent to the tumor tissue, with cancer cells and distant hepatocytes staining negative (Fig. 2i). Functional enrichment analysis showed that genes that interacted with CCL3 were enriched for functions related to RNA metabolism (Fig. 2j). These results suggested that CCL3 is a key chemokine in the interplay between hepatocytes and ICC cells, and could influence RNA metabolic processes such as RNA modification, RNA synthesis, RNA cleavage, and RNA degradation in ICC.

### CCL3 promotes ICC tumor migration and invasion by regulating m^6^A modification via VIRMA

**Fig. 3:**
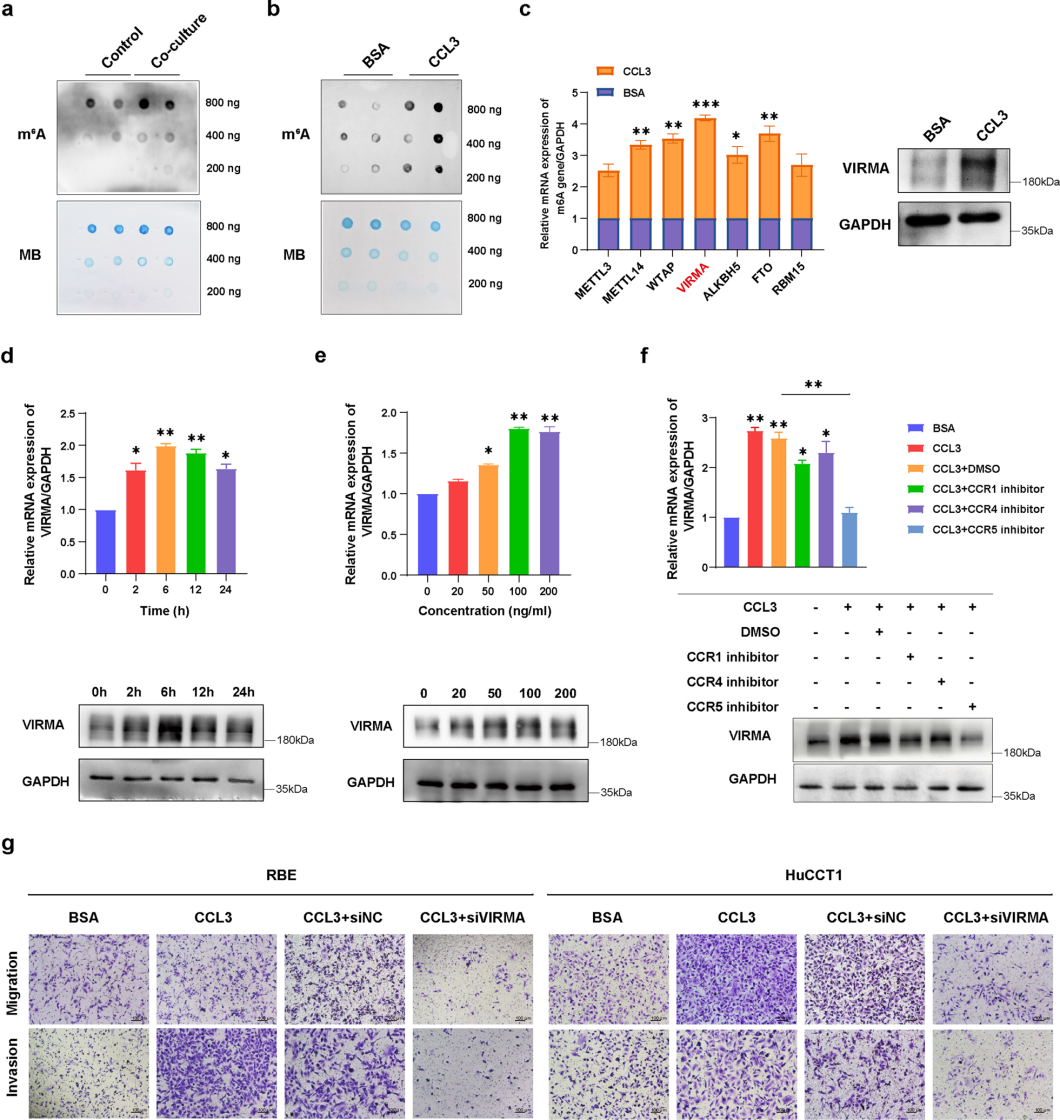
CCL3 promotes tumor migration and invasion by regulating m^6^A modification through VIRMA in ICC. **a** The m^6^A dot blot assay of global m^6^A abundance in mRNA of RBE cells co-cultured with THLE3. **b** The m^6^A dot blot assay of global m^6^A abundance in mRNA of RBE cells stimulated with CCL3 (100 ng/ml) for 6 h. **c** Left: qRT-PCR analysis of m^6^A-related genes mRNA expression in RBE cells stimulated with CCL3. The relative quantification was calculated using the 2^−ΔΔCt^ method and normalized based on GAPDH. Right: western blot analysis of VIRMA protein expression in RBE cells stimulated with CCL3 (100 ng/ml) for 6 h. **d** qRT-PCR and western blot analysis of VIRMA expression in RBE cells stimulated with CCL3 (100 ng/ml) for different times (0, 2, 6, 12, and 24). **e** qRT-PCR and western blot analysis of VIRMA expression in RBE cells stimulated with different concentrations of CCL3 (0, 20, 50, 100, and 200 ng/ml) for 6 h. **f** After treating with CCR1, CCR4, or CCR5 receptor antagonists, qRT-PCR and western blot analysis of VIRMA expression in RBE cells stimulated with CCL3 (100 ng/ml) for 6 h. **g** Pro-metastatic effects of the CCL3/VIRMA pathway were confirmed by transwell assays of RBE cells and HuCCT1 cells with different treatments. Scale bars, 100 μm. *P < 0.05 **P < 0.01, ***P < 0.001

Our dot blot assays demonstrated that m^6^A was among the genes that were shown to interact with CCL3. Global m^6^A abundance was detected in the mRNA of RBE cells co-cultured with THLE3 and stimulated with CCL3. As shown in Fig. 3a, b, the results indicated that the m^6^A in total RNA was upregulated in ICC cells after co-culture with THLE3 and stimulation with CCL3. In addition, the mRNA and protein expression profile of VIRMA, one of the major components of the m^6^A methyltransferase complex, was upregulated in all m^6^A-related writers and readers (Fig. 3c).

Further analysis revealed that VIRMA expression was significantly upregulated in ICC cells treated with 100 ng/ml CCL3 for 6 h (Fig. 3d, e). It was shown that CCL3 could not significantly increase VIRMA expression in the ICC cells treated with CCR5 receptor antagonist (Fig. 3f). These results suggested that the binding of CCL3 to the CCR5 receptor on the membrane of cancer cells promotes ICC migration and invasion by regulating VIRMA, an m^6^A methyltransferase. We then conducted transwell migration and invasion assays of the RBE and HuCCT1 cells with different treatments to assess the role of CCL3/VIRMA pathway on the migratory and invasive ability of the cells. As demonstrated in Fig. 3g, human recombinant cytokine CCL3 effectively promoted migration and invasion of ICC cells, a phenomenon that was inhibited by knockdown of VIRMA, thus confirming the pro-metastatic effects of CCL3/VIRMA pathway in ICC.

### VIRMA promotes ICC development and progression in vitro

**Fig. 4:**
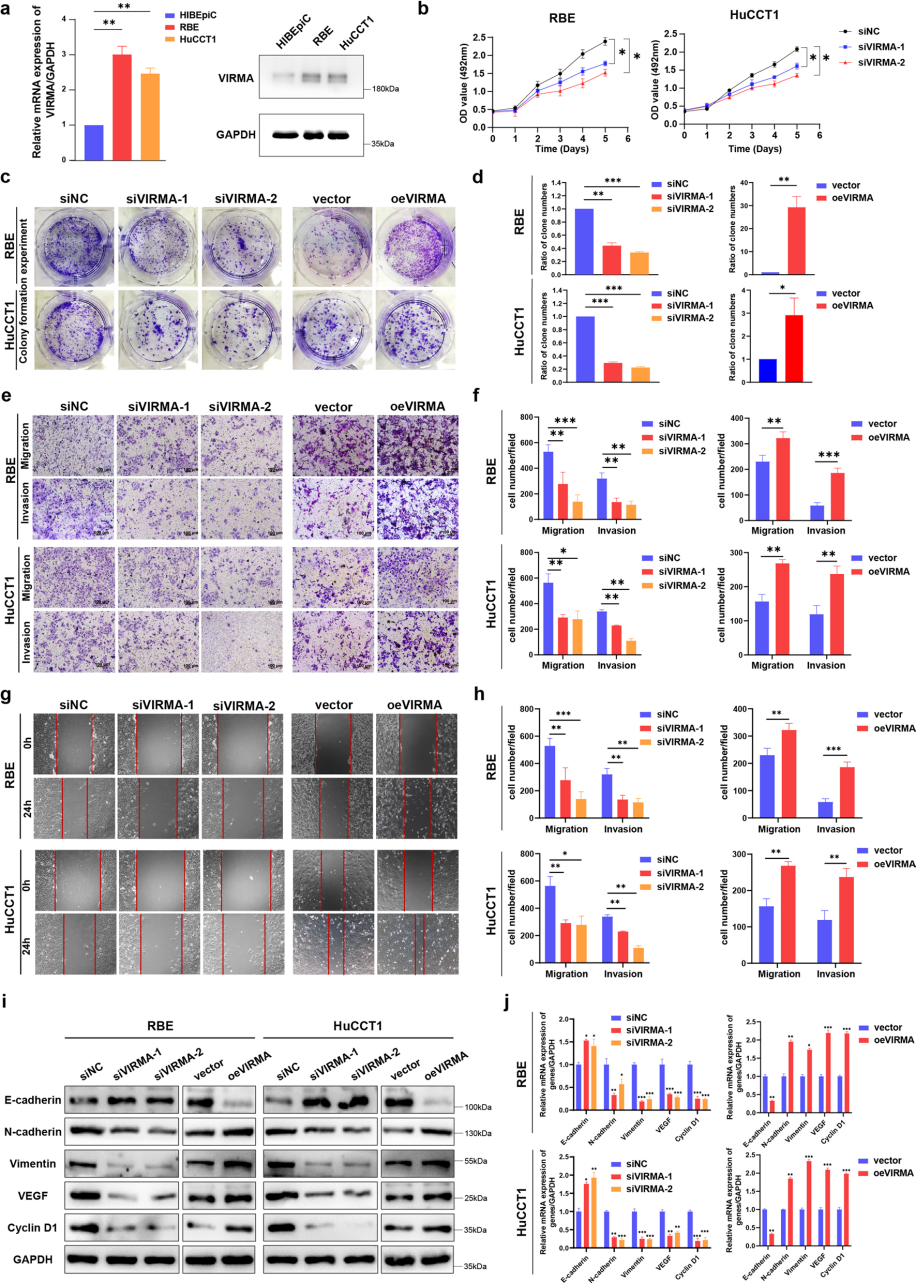
VIRMA promotes ICC proliferation, invasion, and metastasis in vitro. **a** qRT-PCR (up) and western blot (down) analysis of VIRMA expression in ICC cell lines and normal bile duct epithelial cell line HIBEpiC. The relative quantification was calculated using the 2^−ΔΔCt^ method and normalized based on GAPDH. **b** The growth of both RBE and HuCCT1 cells after knockdown or overexpression of VIRMA over 5 days was measured using CCK-8 assays. **c** The growth of cells over 14 days after knockdown or overexpression of VIRMA was measured using colony formation assays. **d** Quantitation of colony formation assays. **e** Transwell migration assay and matrigel invasion assay of RBE cells and HuCCT1 cells after knockdown or overexpression of VIRMA. Scale bars, 100 μm. **f** Quantitation of transwell assays. **g** Wound healing assay of the RBE and HuCCT1 cell lines after knockdown or overexpression of VIRMA. Images of wound repair were taken at 0, 24 h after wounding. **h** Quantitation of wound healing assays. The distance of wound closure was shown by area at 24 h. **i**, **j** The mRNA and protein levels of EMT markers, angiogenic marker VEGF and proliferation marker Cyclin D1 in ICC cells with VIRMA knockdown or overexpression were evaluated by **i** western blot and **j** qRT-PCR. siVIRMA-1 and siVIRMA-2 indicate VIRMA knockdown in ICC cells, while oeVIRMA indicates ICC cells transfected with an overexpressing plasmid. *P < 0.05, **P < 0.01, ***P < 0.001

To examine the roles of VIRMA in the CCA cells, we profiled the VIRMA expression in CCA cell lines using qRT-PCR and Western blot assays. The results illustrated significant upregulation of the VIRMA mRNA and protein levels in ICC cell lines (RBE and HuCCT1) compared with human intrahepatic biliary epithelial cells (HIBEpiC) (Fig. 4a). Thereafter, we performed VIRMA overexpression and knockdown studies in RBE and HuCCT1 cells. The ICC cells were transfected with control siRNA (siNC) or siRNA targeting VIRMA (siVIRMA-1 and siVIRMA-2). On the other hand, the overexpression cell lines were constructed as oeVIRMA while the matched control cell lines were named as vector. The VIRMA expression in the cells was confirmed with qRT-PCR and Western blot (Additional file 3: Fig. S2).

The growth of the RBE and HuCCT1 cells after knockdown or overexpression of the VIRMA was measured using cell counting kit-8 (CCK-8) and colony formation assays. As shown in Fig. 4b–d, there was a declining trend in cell proliferation in RBE and HuCCT1 cells with VIRMA knockdown while overexpression resulted in significantly increased cell proliferation. Moreover, the transwell migration assay demonstrated that downregulation of VIRMA impeded the migratory ability of the ICC cells, while upregulation of VIRMA significantly elevated the migration of the ICC cells. Similarly, the transwell invasion assay showed that the invasive ability of HuCCT1 and RBE cells was significantly suppressed in response to the downregulation of VIRMA, while it was enhanced by VIRMA upregulation (Fig. 4e, f). We then conducted wound healing assays to display the slow migration of cells with VIRMA knockdown, which was in contrast to the increase observed in VIRMA overexpression cells (Fig. 4g, h). In the meantime, we detected the expression of the Epithelial-Mesenchymal Transition (EMT) marker, angiogenic marker vascular endothelial growth factor (VEGF), and proliferation marker Cyclin D1 in ICC cells with VIRMA knockdown or overexpression. As displayed in Fig. 4i, j, the mRNA and protein levels of N-cadherin (mesenchymal marker), Vimentin (mesenchymal marker), VEGF, and Cyclin D1 were significantly positively correlated with VIRMA expression, while there was a prominent inverse correlation between the E-cadherin (epithelial marker) expression and VIRMA expression. These results implied that VIRMA promotes ICC proliferation, invasion, and metastasis in vitro.

**Fig. 5:**
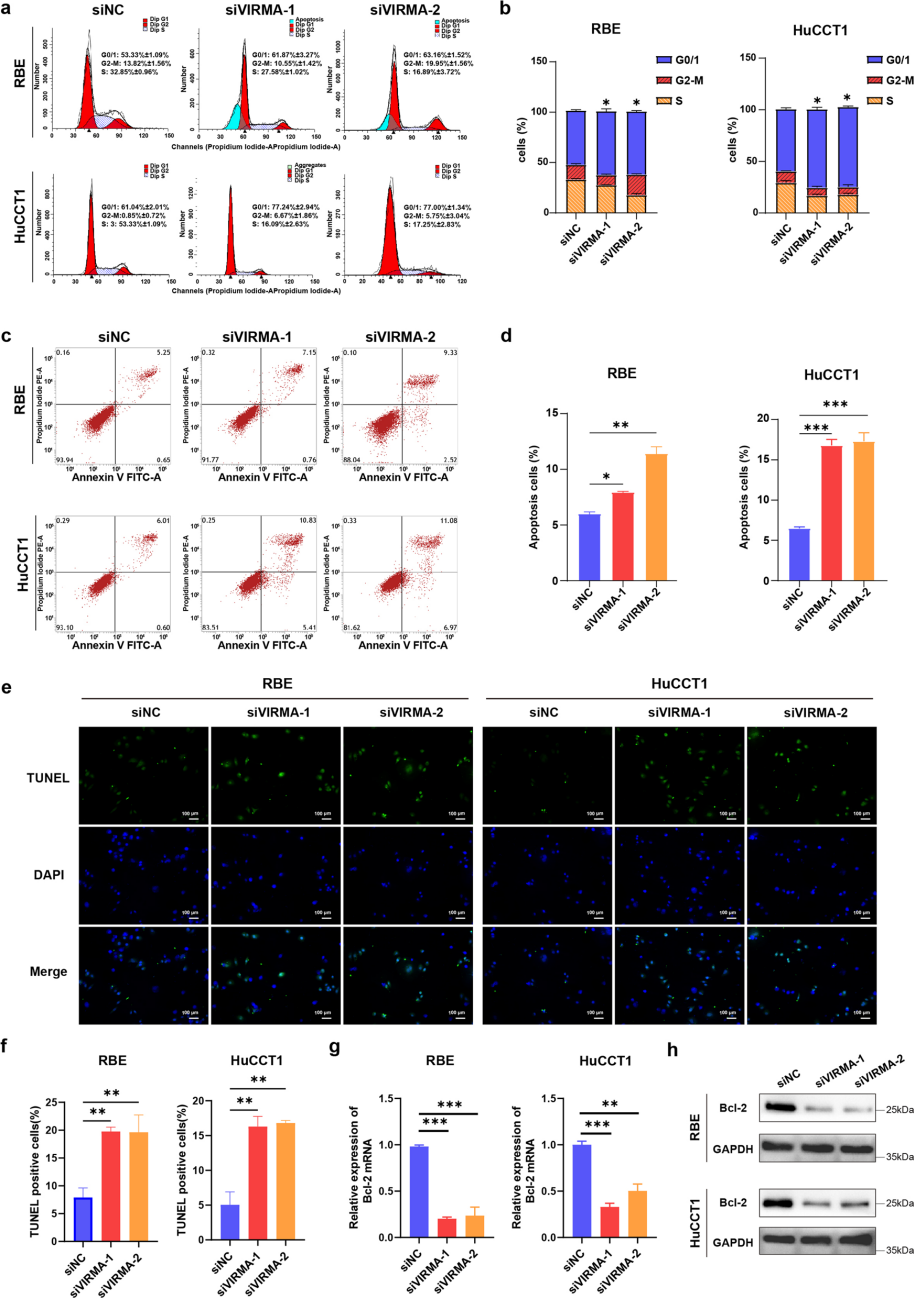
Knockdown of VIRMA induces ICC cell cycle arrest and promotes cell apoptosis. **a** Cell cycle progression was measured using flow cytometry in the RBE and HuCCT1 cells with VIRMA knockdown. **b** Quantitation of cell cycle analysis. **c** Cell apoptosis was measured using flow cytometry in both RBE and HuCCT1 cells with VIRMA knockdown. **d** Quantitation of cell apoptosis analysis. **e** Representative images of TUNEL staining. The nuclei of TUNEL-positive (apoptotic) cells appeared green, indicating apoptotic cells. Scale bars, 200 μm. (**f**) Quantification of TUNEL-positive cells. **g** qRT-PCR analysis of apoptotic marker Bcl-2 mRNA expression in ICC cells with VIRMA knockdown. **h** Western blot analysis of Bcl-2 protein expression in ICC cells with VIRMA knockdown. *P < 0.05, **P < 0.01, ***P < 0.001

Furthermore, we performed cell cycle progression and cell apoptosis analyses. As demonstrated in Fig. 5a, b, VIRMA-deficient cells had a partial cell cycle arrest at the G0/1 transition but a significant increase in the G0/1 phase and a reduction in cells at S and G2-M phases. The apoptosis examination detected by flow cytometry (Fig. 5c, d) and TUNEL assay (Fig. 5e, f) indicated that the percentage of apoptotic cells was significantly elevated in cells transfected with siVIRMA. There appeared an obvious decrease in Bcl-2 expression, the antiapoptotic protein, in ICC cells with down-expression of VIRMA (Fig. 5g, h). From this, we can deduce that downregulation of VIRMA induces ICC cell cycle arrest and promotes cell apoptosis.

### VIRMA promotes ICC proliferation and metastasis in vivo and is correlated with poor prognosis of ICC patients

**Fig. 6:**
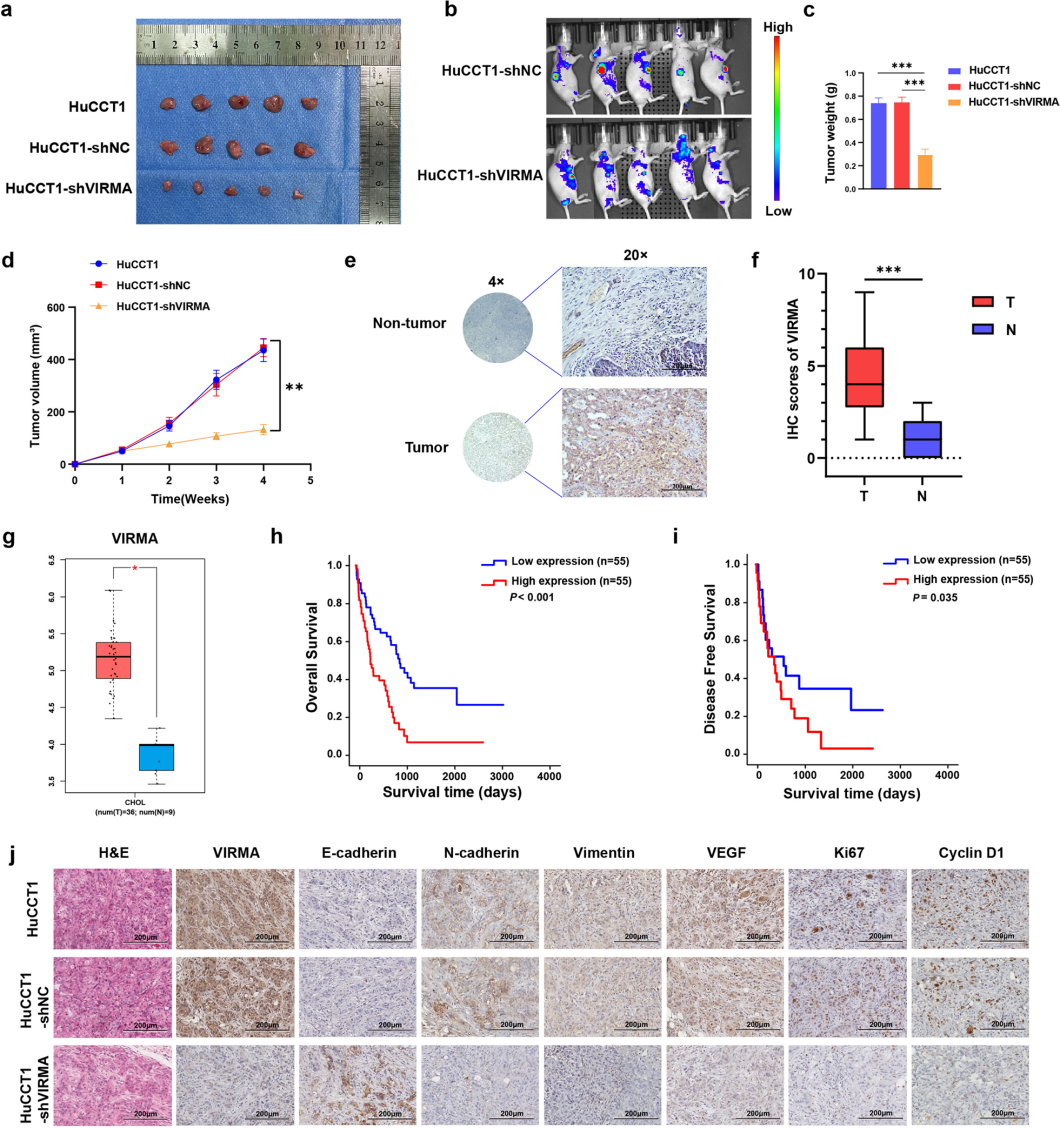
VIRMA promotes ICC proliferation and metastasis in vivo, and is correlated with poor prognosis of ICC patients. Knockdown of VIRMA effectively inhibited ICC subcutaneous tumor growth in nude mice (n = 5). **a** Gross images of ICC subcutaneous tumor. **b** In vivo fluorescence imaging of nude mice bearing ICC tumors. **c** The tumors were extracted and weighed after 4 weeks. **d** The tumor volume was monitored every other day, and tumor growth curves were generated. **e** Representative images of IHC staining for VIRMA protein of 110 ICC tumor tissues and their adjacent normal epithelium tissues. Scale bars, 100 μm. **f** The protein expression level of VIRMA was analyzed in 110 ICC tissues and their paired normal epithelium tissues through IHC staining. **g** The protein expression level of VIRMA was analyzed in ICC tissues and normal epithelium tissues of the TCGA dataset. ***p = 2.21E − 07. The two-tailed *t*-test in paired samples. The box boundaries correspond to the first and third quartiles; whiskers extend to a maximum of 1.5 × the interquartile range. **h**, **i** Kaplan–Meier method with a two-tailed log-rank test was used to plot overall survival (**h**) and disease-free survival (**i**) curves in human ICC specimens (n = 110) with high and low VIRMA expression. The log-rank test was used to compare the survival rate. **j** H&E and IHC staining with an antibody specific for VIRMA, E-cadherin, N-cadherin, Vimentin, VEGF, Ki67 and Cyclin D1 in sections of tumors. *P < 0.05 **P < 0.01, ***P < 0.001

We demonstrated that the downregulation of VIRMA inhibits the proliferation, invasion, and metastasis of ICC cells in vitro. Here, we analyzed the effect of VIRMA in the development of ICC in mice. Stable cells with modified VIRMA expression were subcutaneously injected into the BABL/c nude mice. First, we investigated the effect of VIRMA on the proliferation of ICC. As shown in Fig. 6a–d, VIRMA knockdown (HuCCT1-shVIRMA) led to significantly low tumor growth rate and tumor weight compared with the other groups (HuCCT1: 0.75 ± 0.05 g, HuCCT1-shNC: 0.77 ± 0.02 g, HuCCT1-shVIRMA: 0.28 ± 0.01 g). The H&E staining demonstrated that VIRMA knockdown significantly reduced the tumor density and differentiation degree of the ICC. On the other hand, immunohistochemical staining showed that the expression of tumor progression markers such as N-cadherin, Vimentin, VEGF, Ki67, and Cyclin D1 was decreased along with the downregulation of VIRMA, except E-cadherin exhibited a contrary tendency (Fig. 6j).

In parallel, we analyzed the expression of VIRMA in ICC patients using the TCGA dataset and showed that the VIRMA expression was remarkably increased in CCA tissues (Fig. 6g). Moreover, immunohistochemical staining in 110 human ICC patients and normal adjacent tissues revealed that VIRMA expression was significantly higher in ICC cancer compared to the adjacent counterparts (Fig. 6e, f). In addition, we conducted a Kaplan–Meier survival analysis to investigate the correlation between VIRMA expression and ICC patient prognosis (Fig. 6h, i). The results demonstrated that ICC patients with high VIRMA expression had poorer overall survival (OS) and disease-free survival (DFS) (*P < 0.05). Taken together, these findings suggested that the higher expression of VIRMA was associated with adverse prognosis in ICC patients.

### SIRT1 was selected as a downstream target of VIRMA-mediated m^6^A modification

**Fig. 7:**
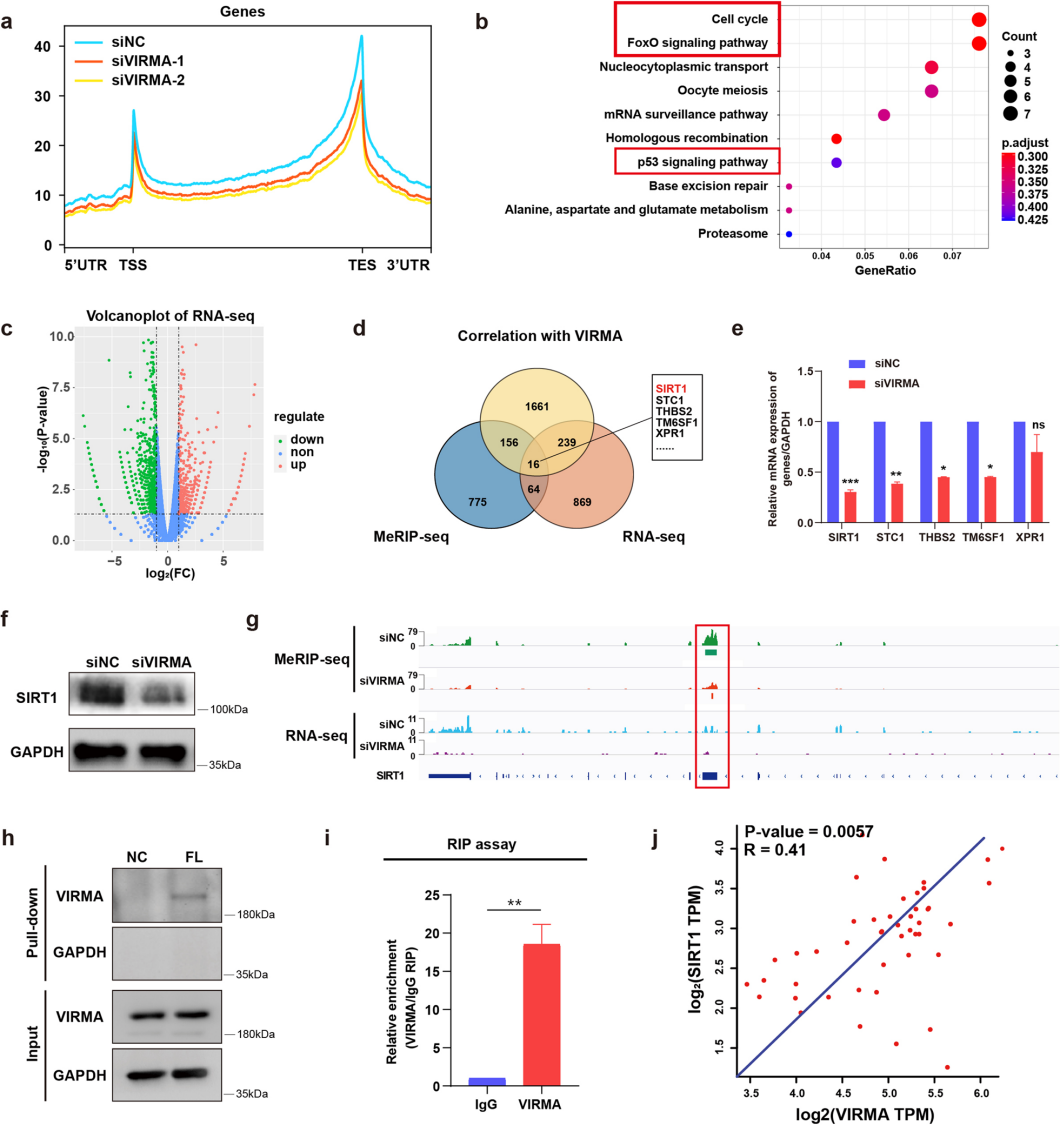
SIRT1 was selected as a downstream target of VIRMA-mediated m^6^A modification. **a** The proportion analysis for specific m^6^A binding sites in MeRIP-seq. TSS, transcription start site; TES, transcription end site. **b** KEGG analysis of down-regulated genes identified for MeRIP-seq. **c** Volcano figure of RNA-seq analysis. **d** Differential genes are generated by the intersection of sequencing results. **e** qRT-PCR analysis of mRNA expression of the above-mentioned differential genes in ICC cell line with VIRMA knockdown. **f** Western blot analysis of SIRT1 protein expression in ICC cell line with VIRMA knockdown. **g** IGV of SIRT1. **h**, **i** RNA pull-down analysis (**h**) and RIP assays (**i**) were employed to determine the interaction of SIRT1 and VIRMA. **j** Correlation analysis between SIRT1 and VIRMA in TCGA ICC dataset. *P < 0.05, **P < 0.01, ***P < 0.001

To identify the target genes of VIRMA, MeRIP-seq and RNA-seq were performed in stable VIRMA downregulation ICC cells. According to the results of the MeRIP-seq, it was found that more than 80% of m^6^A binding sites were located in the protein-coding region (CDS region) and were enriched in the 5′ untranslated region (5′ UTR) as well as 3′ untranslated region (3′ UTR). Further analysis showed that the proportion of specific m^6^A binding sites in the 3′UTR was reduced in the siVIRMA group as compared with that in the siNC group and this was consistent with findings that VIRMA was mainly involved in regulating m^6^A modification in the 3′UTR region of mRNA (Fig. 7a).

The results of the Motif for the m^6^A binding site indicated that the most significant m^6^A Motif was GATG (Additional file 3: Fig. S3c), which provided a basis for subsequent screening of the downstream targets. Network enrichment analysis was then conducted according to MeRIP-seq results and it was identified that the cell cycle pathway, as well as the Foxo signaling pathway, were significantly associated with the oncogenic activities of VIRMA (Fig. 7b). Kyoto Encyclopedia of Genes and Genomes (KEGG) and gene ontology (GO) enrichment analysis were carried out using gene set enrichment analysis (GSEA) (Additional file 3: Fig. S3). Results of the RNA-seq showed that 1188 transcripts were markedly downregulated on VIRMA knockdown. Further, MeRIP-seq presented that m^6^A peaks of 1011 transcripts exhibited decreased abundance. In addition, it was found that 2072 genes were significantly correlated with VIRMA in TCGA dataset (Fig. 7c).

Intriguingly, results of the transcriptome-sequencing, MeRIP-seq in the genes selected in the TCGA dataset revealed that 16 transcripts were overlapped (Fig. 7d). Further, it was noted that SIRT1 showed the most pronounced difference and was reported to be close to tumor progression among the candidate genes. The m^6^A usually happens in RRACH (R = G or A, H = A, C or U) consensus sequence, results of MeRIP-seq in the present study showed that an m^6^A peak was detected around the stop codon of SIRT1 mRNA in non-target control siRNA cells and were all decreased upon VIRMA knockdown (Fig. 7g). The SIRT1 mRNA was also significantly downregulated in VIRMA-knockdown ICC cells both at mRNA and protein levels (Fig. 7e, f). Furthermore, the RNA pull-down and RIP assays were also conducted to verify the positive association between SIRT1 and VIRMA (Fig. 7h, i). The correlation analysis between SIRT1 and VIRMA based on the TCGA ICC dataset in Fig. 7j reached a similar conclusion. Therefore, a preliminary conclusion was drawn that SIRT1 was selected for further studies as a candidate target of VIRMA-mediated m^6^A modification.

### Downregulation of SIRT1 repressed tumor progression of ICC cells in vitro

**Fig. 8:**
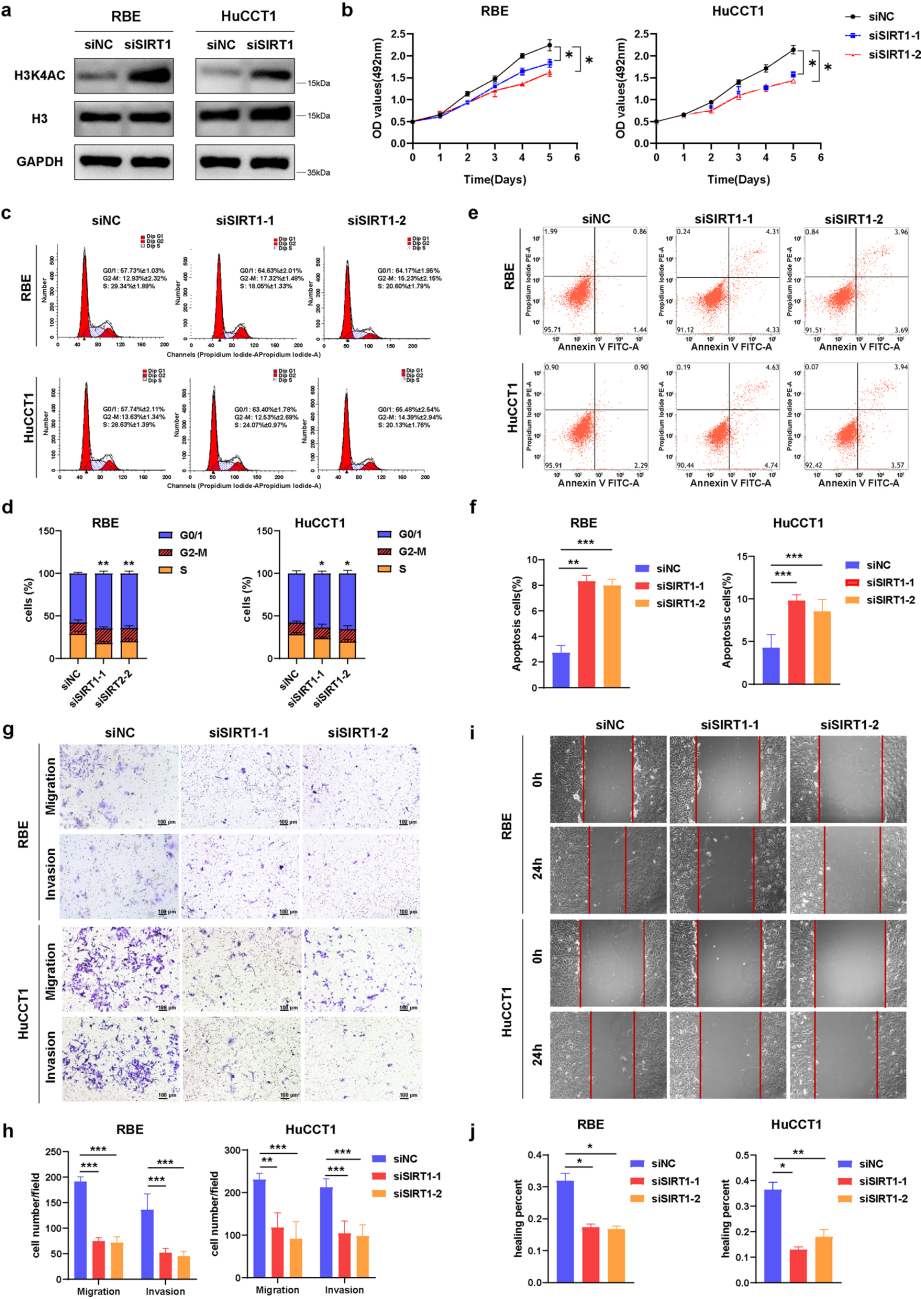
Downregulation of SIRT1 repressed tumor progression of ICC cells in vitro. **a** Western blot analysis of H3K4ac protein level in ICC cell lines after SIRT1 knockdown. **b** Cell growth by CCK-8 assays of ICC cells with SIRT1 knockdown. **c** Cell cycle progression was measured by flow cytometry of ICC cells with SIRT1 knockdown. **d** Quantitation of cell cycle analysis. **e** Cell apoptosis was measured by flow cytometry of ICC cells with SIRT1 knockdown. **f** Quantitation of cell apoptosis analysis. **g** Transwell migration assay and matrigel invasion assay of ICC cells with SIRT1 knockdown. Scale bars, 100 μm. **h** Quantitation of transwell assays. **i** Wound healing assay of ICC cells with SIRT1 knockdown. Images of wound repair were taken at 0, 24 h after wounding. **j** Quantitation of wound healing assays. The distance of wound closure was shown by area at 24 h. siSIRT1-1 and siSIRT1-2 indicate SIRT1 knockdown in ICC cells. *P < 0.05, **P < 0.01, ***P < 0.001

As a kind of histone deacetylase, SIRT1 may regulate the chromatin acetylation process. Therefore, we observed that the H3K4 acetylation (H3K4ac) protein level was decreased after SIRT1 knockdown in both RBE and HuCCT1 cells (Fig. 8a). Cell viability was determined by CCK8 assays, from which we observed that downregulation of SIRT1 notably inhibited tumor cell growth in ICC (Fig. 8b). Moreover, we conducted cell cycle progression analysis and cell apoptosis assay of RBE and HuCCT1 cells after SIRT1 knockdown. As illustrated in Fig. 8c, d, depletion of SIRT1 induced tumor cell arrest in the G0/1 phase. And it also markedly increased cell apoptosis in ICC (Fig. 8e, f). Transwell assays (Fig. 8g, h) and wound healing assays (Fig. 8i, j) were performed through SIRT1 knockdown to evaluate the role of SIRT1 in the regulation of migration and invasion ability in the ICC cells. Results of the current study showed that the downregulation of SIRT1 significantly decreased the migration and invasion ability of HuCCT1 and RBE cells compared with the control group.

### CCL3/VIRMA/SIRT1 pathway accelerates the ICC malignant process

**Fig. 9:**
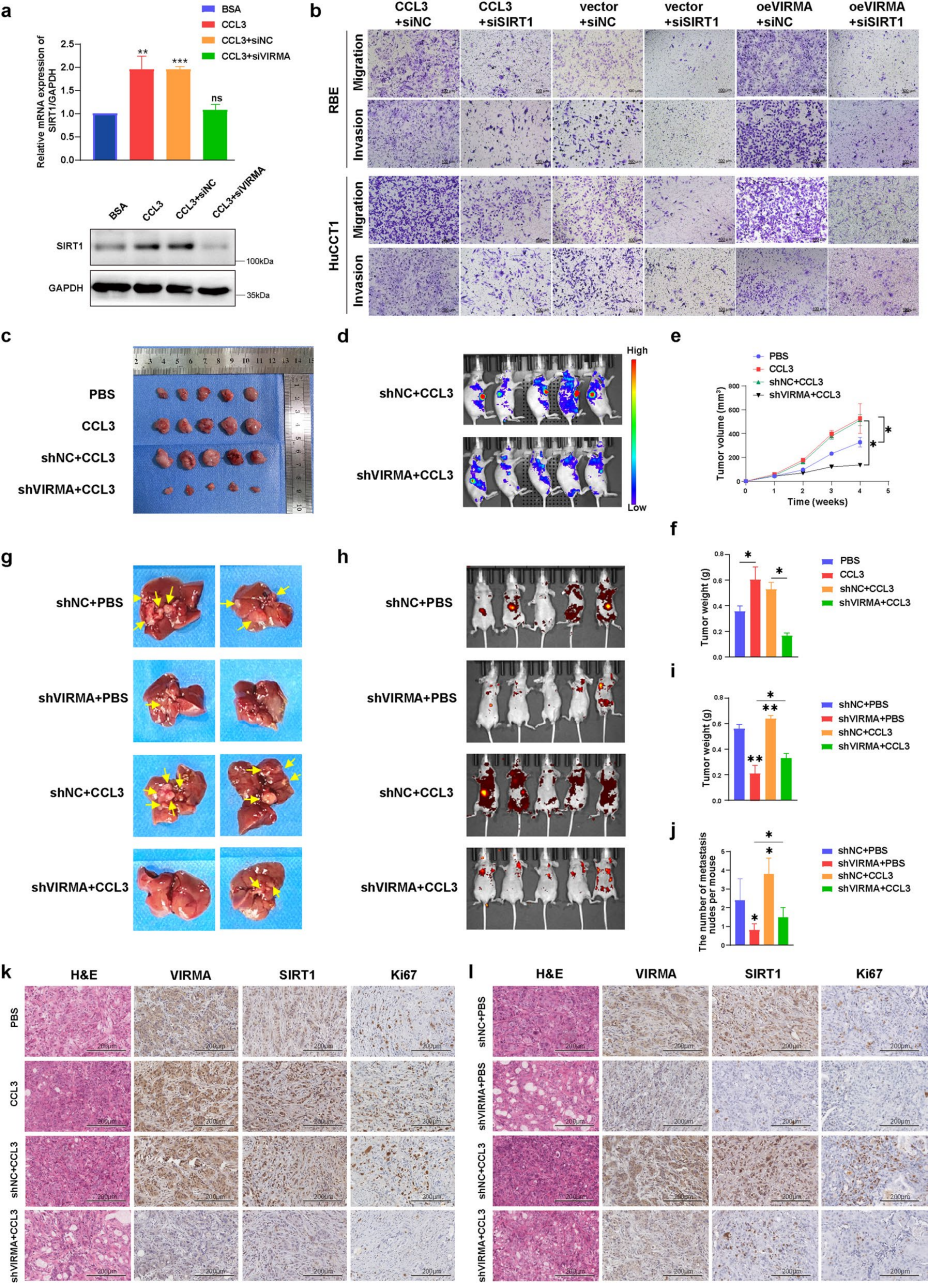
CCL3/VIRMA/SIRT1 pathway accelerates the ICC malignant process. **a** qRT-PCR analysis (up) and western blot analysis (down) of SIRT1 expression in rescue experiment. **b** Transwell migration assay and matrigel invasion assay of RBE cells and HuCCT1 cells in rescue experiment. Scale bars, 100 μm. **c–f** Intratumoral injections of CCL3 effectively promoted ICC subcutaneous tumor growth in nude mice, whereas the knockdown of VIRMA inhibited the progression. **c** Gross images of ICC subcutaneous tumor. **d** In vivo fluorescence imaging of nude mice bearing ICC tumors. **e** The tumor volume was monitored on daily basis, and tumor growth curves were generated. **f** The tumors were extracted and weighed after 4 weeks. **g–j** Stimulation of CCL3 effectively promoted ICC tumor liver metastasis in the orthotopic xenograft nude mice model, whereas the knockdown of VIRMA inhibited this progression. **g** Representative images of metastasis in the livers. **h** In vivo fluorescence imaging of orthotopic xenograft nude mouse model bearing ICC tumors. **i** The tumors were extracted and weighed after 8 weeks. **j** Quantification of the liver metastasis. **k** Sections of ICC subcutaneous tumor and **l** orthotopic xenograft tumor were stained with antibodies through IHC staining. The tumor tissues were stained with an antibody specific for VIRMA, SIRT1, or Ki67. Scale bars, 200 μm. *P < 0.05 **P < 0.01, ***P < 0.001

It was evident that the CCL3 can abolish the effect of VIRMA knockdown in SIRT1 expression (Fig. 9a, b and Additional file 3: Fig. S4) during the rescue experiments in vitro. Therefore, SIRT1 is a downstream target of CCL3/VIRMA in ICC which served as an oncogene to promote migration and invasion of ICC cells.

To explore the effect of the CCL3/VIRMA/SIRT1 pathway on the ICC malignant process, we constructed subcutaneous and orthotopic tumor models in nude mice.

We first injected different cells (HuCCT1 + PBS, HuCCT1 + CCL3, HuCCT1-shNC + CCL3, and HuCCT1-shVIRMA + CCL3) subcutaneously into nude mice. As shown in Fig. 9c–f, tumor growth of HuCCT1 ICC cells was significantly faster than that in the PBS group after CCL3 treatment, while HuCCT1 cells with VIRMA knockdown grew slower even after receiving CCL3 stimulation. Our data showed that the tumor weight in the CCL3 treatment group was significantly higher than that in the PBS group, while the VIRMA knockdown group was not affected by CCL3 treatment, and the tumor weight was significantly lower than that in the control group (HuCCT1 + PBS: 0.81 ± 0.02 g, HuCCT1 + CCL3: 1.45 ± 0.08 g, HuCCT1-shNC + CCL3: 1.53 ± 0.04 g, and HuCCT1-shVIRMA + CCL3: 0.27 ± 0.02 g). The immunohistochemistry analyses confirmed that the expression of VIRMA, SIRT1, and Ki67 was upregulated in the tumor tissues with CCL3 stimulation, and it was also reversed in the tumor tissues with VIRMA down-expression (Fig. 9k). Together, these data demonstrated that CCL3 promotes the growth of ICC cells by regulating VIRMA/SIRT1 in ICC subcutaneous tumor mice model.

We further showed that CCL3 promoted the metastasis of intrahepatic bile duct cancer cells by regulating VIRMA/SIRT1 pathway in the orthotopic ICC tumor model. The liver metastasis was significantly enhanced with CCL3 stimulation, whereas the progression was significantly inhibited by VIRMA knockdown (Fig. 9g–j). Furthermore, IHC staining of the nude mice tissues (Fig. 9l) also verified that SIRT1 was positively correlated with VIRMA expression. With CCL3 stimulation, the Ki67 was up-regulated in the tumor tissues and it was also reversed in the tumor tissues with VIRMA down-expression. Consistent with the above observations, it was evident that the CCL3 had positive effects on tumor growth and liver metastasis of ICC and the progression could be abolished through VIRMA knockdown.

In conclusion, the results of the current study confirmed that CCL3/VIRMA/SIRT1 pathway promotes ICC proliferation and tumor metastasis in vivo.

## Discussion

Metastasis is the main clinical challenge in ICC. Tumor metastasis is affected by the TME, a unique environment that arises because of tumor progression. Most studies on the mechanism of ICC metastasis have focused on the analysis of the ICC cells, but only a few have assessed the role of other cells in TME. The liver cancer microenvironment is composed of cells such as immune cells, fibroblasts, and liver parenchymal cells as well as extracellular matrix components [33]. Previous studies have demonstrated that ICC development is affected by the liver microenvironment, which promotes tumor proliferation, infiltration, and metastasis by releasing cytokines and regulating the transcription and translation of tumor-related genes [34, 35].

In this study, we showed that the co-culture of the ICC cells with hepatocytes significantly enhanced the migration and invasion ability of the cells**.** Moreover, to clarify the molecular interaction between hepatocytes and ICC cells, we performed a cytokine microarray analysis and showed that CCL3 is the main chemokine that is involved in the interaction. CCL3, also referred to as macrophage inflammatory protein-1 (MIP-1α), belongs to the C–C chemokine family. Studies have shown that CCL3 contributes to the development and progression of various malignancies, such as esophageal cancer, breast cancer, osteosarcoma, leukemia, and multiple myeloma [36]. As expected, our data demonstrated that recombinant human CCL3 obviously promoted migration and invasion of the ICC cells.

Thereafter, we analyzed the mechanisms of CCL3 activity in the communication between hepatocytes and ICC. Functional enrichment analysis showed that the genes that interacted with CCL3 were enriched for functions related to RNA metabolism. We further demonstrated that chemical modification occurs at the RNA level, which affects gene-regulatory networks at the epigenetic level. Previous studies demonstrated an association between extracellular signals such as cytokines or chemokines and m^6^A modification [14, 15]. The m^6^A RNA modification has been identified as a source of epigenetic regulation in DNA modification. The m^6^A modification accounts for more than 80% of RNA modification and is a reversible and dynamic process regulated by m^6^A methyltransferase, demethylases, and readers [37]. m^6^A methylation of mRNA has been reported to play significant roles in many RNA functions, such as RNA splicing, RNA processing, translation regulation, and RNA decay [18–20, 38, 39]. However, data on the role of m^6^A modification in ICC remains limited. In this study, there was an increased m^6^A modification in ICC cells co-cultured with hepatocytes, which was potentially affecting cancer metastasis. We showed that CCL3 was the key factor mediating the interaction between the liver microenvironment and ICC cells. Subsequently, we characterized methyltransferase VIRMA and showed that it is the aberrant m^6^A modification in ICC cells co-cultured with hepatocytes.

VIRMA is an important member of the m^6^A methyltransferase complex and is mainly located in the nucleosome and cytoplasm. As a key regulator of m^6^A methylation, VIRMA was shown to form a complex with WTAP/METTL3/METTL14 heterodimer to catalyze m^6^A formation [25, 40]. Recent studies have shown that VIRMA is upregulated in several malignancies such as breast cancer [32], lung cancer [41], germ cell tumor [42], and liver cancer [43], and is involved in tumor proliferation and metastasis. So far, however, there are limited studies that have evaluated the biological function and mechanism of VIRMA-mediated m^6^A modification in ICC. In this study, we showed that VIRMA was significantly upregulated in ICC tissues, which correlated with poor prognosis and thus regarded as a prognostic factor for tumor recurrence in ICC. Moreover, our in vitro and in vivo assays demonstrated that VIRMA promotes malignant development and progression of ICC, and its expression showcased a significant correlation with various metastatic, angiogenic and proliferation gene markers. These findings suggested that VIRMA may function as a tumor-promoting gene and might be a potential clinical prognostic biomarker and therapeutic target for ICC.

Analysis of the RNA-Seq and MeRIP-Seq data identified SIRT1 (Sirtuin type 1), as a crucial downstream target of VIRMA. Our RNA pull-down and RIP assays demonstrated that SIRT1 was positively regulated by VIRMA and modified by VIRMA-mediated m^6^A methylation. Thereafter, we conducted GO and KEGG enrichment analyses to explore the major biological functions and downstream signaling pathways of the genes from the RNA-Seq and MeRIP-Seq analyses, respectively. SIRTI, a nicotinamide adenine dinucleotide (NAD+)-dependent histone deacetylase, plays important roles in oncogenesis via transcription, translation, and post-translational modifications [44]. SIRT1 was shown to be a potential tumor promoter based on its role in negatively regulating many tumors suppressors [45, 46]. In sync with previous studies, our findings showed that SIRT1 facilitates the proliferation and migration of tumor cells in ICC. As previously demonstrated, SIRT1 modulates various pathways such as the p53 signaling pathway [47, 48] and FoxO signaling pathway [44] to control cell cycle, metabolism, proliferation, differentiation, and epigenetics. These data agree with that obtained by GO and KEGG enrichment analysis of MeRIP-seq. Therefore, CCL3/VIRMA/SIRT1 axis may be one of the important mechanisms underlying tumor evolution and metastasis and is a potential new therapeutic target for ICC.

In our study, we only employed the m^6^A dot blot assay to quantify the m^6^A levels of ICC, and showed that SIRT1 is a downstream target of VIRMA. Thus, there is a need for further studies which would take the following variables into account: (1) examine the m^6^A levels of ICC using a colorimetric strategy or liquid chromatography-mass spectrometry (LC–MS), (2) detect whether VIRMA functions independently of its m^6^A catalytic activity in cancer progression, and (3) develop a peptide inhibitor to target VIRMA domain and explore whether it may be beneficial in the treatment of ICC.

In conclusion, our study highlights the critical role of VIRMA-mediated m^6^A modification in ICC progression and metastasis. Mechanistically, we demonstrated that CCL3 is secreted by hepatocytes and may promote metastasis of ICC cells by regulating m^6^A methylation. The regulation of m^6^A methylation is mediated by VIRMA, which epigenetically promotes SIRT1 expression through an m^6^A methylation-dependent mechanism. Our results suggested that the interaction between hepatocytes and ICC cells might offer a possible interventional target for ICC. Besides, the m^6^A modification on tumor metastasis will contribute to further studies that would explore molecular mechanisms and identify efficient treatment strategies against ICC.

## Supplementary Information

**Additional file 1: Table S1.** Primers used in qRT-PCR analysis.

**Additional file 2: Table S2.** siRNA sequences.

**Additional file 3: Figure S1.** Functional enrichment analysis of cytokine antibody array. **Figure S2**. Validation of VIRMA knockdown and overexpression in ICC cells. (a) qRT-PCR (up) and western blot (down) analysis of VIRMA expression in VIRMA-knockdown ICC cells. siVIRMA-1 and siVIRMA-2 indicated VIRMA knockdown in both RBE and HuCCT1 cells. (b) qRT-PCR (up) and western blot (down) analysis of VIRMA expression in VIRMA-overexpression ICC cells. oeVIRMA indicated RBE cells and HuCCT1 cells transfected with an overexpressing plasmid. The relative quantification was calculated using the 2^−ΔΔCt^ method and normalized based on GAPDH.** Figure S3.** MeRIP-seq and RNA-seq analysis. (a) GO analysis of down-regulated genes identified for MeRIP-seq. (b) Motif analysis. (c) The peak annotation for m^6^A signal enrichment in MeRIP-seq. (d) GO analysis of down-regulated genes identified for RNA-seq. (e) KEGG analysis of down-regulated genes identified for RNA-seq. (f) GO analysis of up-regulated genes identified for RNA-seq. (g) KEGG analysis of up-regulated genes identified for RNA-seq. **Figure S4. **Quantification of rescue experiments.

**Additional file 4:** Additional methods and experimental details.

## Data Availability

The dataset supporting the conclusions of this article is included within the article and its additional files.

## References

[1] Razumilava N, Gores GJ (2014). Cholangiocarcinoma. Lancet.

[2] Rizvi S, Khan SA, Hallemeier CL, Kelley RK, Gores GJ (2018). Cholangiocarcinoma-evolving concepts and therapeutic strategies. Nat Rev Clin Oncol.

[3] Banales JM (2020). Cholangiocarcinoma 2020: the next horizon in mechanisms and management. Nat Rev Gastroenterol Hepatol.

[4] Kelley RK, Bridgewater J, Gores GJ, Zhu AX (2020). Systemic therapies for intrahepatic cholangiocarcinoma. J Hepatol.

[5] Hui L, Chen Y (2015). Tumor microenvironment: sanctuary of the devil. Cancer Lett.

[6] Matsumoto T, Wakefield L, Tarlow BD, Grompe M (2020). In vivo lineage tracing of polyploid hepatocytes reveals extensive proliferation during liver regeneration. Cell Stem Cell.

[7] de Palma M, Biziato D, Petrova TV (2017). Microenvironmental regulation of tumour angiogenesis. Nat Rev Cancer.

[8] Quail DF, Joyce JA (2013). Microenvironmental regulation of tumor progression and metastasis. Nat Med.

[9] Fabris L, Sato K, Alpini G, Strazzabosco M (2021). The tumor microenvironment in cholangiocarcinoma progression. Hepatology.

[10] Gentilini A (2012). Role of the stromal-derived factor-1 (SDF-1)-CXCR4 axis in the interaction between hepatic stellate cells and cholangiocarcinoma. J Hepatol.

[11] Yang X (2016). FAP promotes immunosuppression by cancer-associated fibroblasts in the tumor microenvironment via STAT3-CCL2 signaling. Can Res.

[12] Chow MT, Luster AD (2014). Chemokines in cancer. Cancer Immunol Res.

[13] Nagarsheth N, Wicha MS, Zou W (2017). Chemokines in the cancer microenvironment and their relevance in cancer immunotherapy. Nat Rev Immunol.

[14] Chang G, Leu JS, Ma L, Xie K, Huang S (2019). Methylation of RNA N6-methyladenosine in modulation of cytokine responses and tumorigenesis. Cytokine.

[15] Xie Z (2021). TNF-α-mediated m^6^A modification of ELMO1 triggers directional migration of mesenchymal stem cell in ankylosing spondylitis. Nat Commun.

[16] Bertero A (2018). The SMAD2/3 interactome reveals that TGFβ controls m 6 A mRNA methylation in pluripotency. Nature.

[17] Lasman L, Hanna JH (2018). M^6^A deposition: A boost from TGFβ. Cell Res.

[18] Liu N, Pan T (2016). N6-methyladenosine–encoded epitranscriptomics. Nat Struct Mol Biol.

[19] Zhao BS, Roundtree IA, He C (2017). Post-transcriptional gene regulation by mRNA modifications. Nat Rev Mol Cell Biol.

[20] Meyer KD, Jaffrey SR (2014). The dynamic epitranscriptome: N6-methyladenosine and gene expression control. Nat Rev Mol Cell Biol.

[21] Xiang Y (2017). RNA m^6^A methylation regulates the ultraviolet-induced DNA damage response. Nature.

[22] Wen J (2018). Zc3h13 regulates nuclear RNA m^6^A methylation and mouse embryonic stem cell self-renewal. Mol Cell.

[23] Su R (2020). Targeting FTO suppresses cancer stem cell maintenance and immune evasion. Cancer Cell.

[24] He L (2019). Functions of N6-methyladenosine and its role in cancer. Mol Cancer.

[25] Wang T, Kong S, Tao M, Ju S (2020). The potential role of RNA N6-methyladenosine in cancer progression. Mol Cancer.

[26] Wang Q (2020). METTL3-mediated m 6 A modification of HDGF mRNA promotes gastric cancer progression and has prognostic significance. Gut.

[27] Chen M (2018). RNA N6-methyladenosine methyltransferase-like 3 promotes liver cancer progression through YTHDF2-dependent posttranscriptional silencing of SOCS2. Hepatology.

[28] Liu T (2021). The m^6^A reader YTHDF1 promotes ovarian cancer progression via augmenting EIF3C translation. Nucleic Acids Res.

[29] Chang G (2020). YTHDF3 induces the translation of m^6^A-enriched gene transcripts to promote breast cancer brain metastasis. Cancer Cell.

[30] Chen H (2021). RNA N6-methyladenosine methyltransferase METTL3 facilitates colorectal cancer by activating the m^6^A-GLUT1-mTORC1 axis and is a therapeutic target. Gastroenterology.

[31] Chen X (2020). METTL14-mediated N6-methyladenosine modification of SOX4 mRNA inhibits tumor metastasis in colorectal cancer. Mol Cancer.

[32] Qian JY (2019). KIAA1429 acts as an oncogenic factor in breast cancer by regulating CDK1 in an N6-methyladenosine-independent manner. Oncogene.

[33] Brodt P (2016). Role of the microenvironment in liver metastasis: from pre- to prometastatic niches. Clin Cancer Res.

[34] Fabris L, Sato K, Alpini G, Strazzabosco M (2021). The tumor microenvironment in cholangiocarcinoma progression. Hepatology.

[35] Gentilini A, Pastore M, Marra F, Raggi C (2018). The role of stroma in cholangiocarcinoma: the intriguing interplay between fibroblastic component, immune cell subsets and tumor epithelium. Int J Mol Sci.

[36] Korbecki J, Grochans S, Gutowska I, Barczak K, Baranowska-Bosiacka I (2020). CC chemokines in a tumor: a review of pro-cancer and anti-cancer properties of receptors CCR5, CCR6, CCR7, CCR8, CCR9, and CCR10 Ligands. Int J Mol Sci.

[37] Meyer KD, Jaffrey SR (2017). Rethinking m 6 A readers, writers, and erasers. Annu Rev Cell Dev Biol.

[38] Fu Y, Dominissini D, Rechavi G, He C (2014). Gene expression regulation mediated through reversible m 6 A RNA methylation. Nat Rev Genet.

[39] Roundtree IA, Evans ME, Pan T, He C (2017). Dynamic RNA modifications in gene expression regulation. Cell.

[40] Deng X (2018). RNA N6-methyladenosine modification in cancers: current status and perspectives. Cell Res.

[41] Xu Y (2021). VIRMA contributes to non-small cell lung cancer progression via N6-methyladenosine-dependent DAPK3 post-transcriptional modification. Cancer Lett.

[42] Miranda-Gonçalves V (2021). The component of the m^6^A writer complex VIRMA is implicated in aggressive tumor phenotype, DNA damage response and cisplatin resistance in germ cell tumors. J Exp Clin Cancer Res.

[43] Lan T (2019). KIAA1429 contributes to liver cancer progression through N6-methyladenosine-dependent post-transcriptional modification of GATA3. Mol Cancer.

[44] Dasgupta A (2020). SIRT1-NOX4 signaling axis regulates cancer cachexia. J Exp Med.

[45] Sun L (2013). A SUMOylation-dependent pathway regulates SIRT1 transcription and lung cancer metastasis. JNCI J Natl Cancer Inst.

[46] Miller JJ (2021). Sirtuin activation targets IDH-mutant tumors. Neuro Oncol.

[47] Zhang ZY (2015). SIRT1 regulates oncogenesis via a mutant p53-dependent pathway in hepatocellular carcinoma. J Hepatol.

[48] Ong ALC, Ramasamy TS (2018). Role of Sirtuin1-p53 regulatory axis in aging, cancer and cellular reprogramming. Ageing Res Rev.

