# Supplementary material for: CCL3 secreted by hepatocytes promotes the metastasis of intrahepatic cholangiocarcinoma by VIRMA-mediated N6-methyladenosine (m6A) modification
Source: J Transl Med. 2023 Jan 23;21:43. doi: 10.1186/s12967-023-03897-y (PMC9869516; doi:10.1186/s12967-023-03897-y)
Supplement: Supplementary file 1 — Additional file 1: Table S1. Primers used in qRT-PCR analysis. [file 12967_2023_3897_MOESM1_ESM.docx]

**Table S1 | Primers used in qRT-PCR analysis**

| **Target** | **Primer Sequences (5' - 3')** | |
| --- | --- | --- |
| **CCL3** | Forward | TGCAACCAGTTCTCTGCATC |
|  | Reverse | TTTCTGGACCCACTCCTCAC |
| **CCL23** | Forward | CATCTCCTACACCCCACGAAG |
|  | Reverse | GGGTTGGCACAGAAACGTC |
| **CCR1** | Forward | GCAGCCTTCACTTTCCTCAC |
|  | Reverse | AGAGGAAGGGGAGCCATTTA |
| **CCR4** | Forward | TCTCGCCAAGACACTGAACAG |
|  | Reverse | GGCCCTGCATTCCTCAAGAAG |
| **CCR5** | Forward | TAGTCATCTTGGGGCTGGTC |
|  | Reverse | TGAACTTCTCCCCGACAAAG |
| **VIRMA** | Forward | AAGTGCCCCTGTTTTCGATAG |
|  | Reverse | ACCAGACCATCAGTATTCACCT |
| **SIRT1** | Forward | TAGCCTTGTCAGATAAGGAAGGA |
|  | Reverse | ACAGCTTCACAGTCAACTTTGT |
| **GAPDH** | Forward | TCAACGGATTTGGTCGTATTGGGCG |
|  | Reverse | CTCGCTCCTGGAAGATGGTGATGGG |
