# Supplementary material for: CCL3 secreted by hepatocytes promotes the metastasis of intrahepatic cholangiocarcinoma by VIRMA-mediated N6-methyladenosine (m6A) modification
Source: J Transl Med. 2023 Jan 23;21:43. doi: 10.1186/s12967-023-03897-y (PMC9869516; doi:10.1186/s12967-023-03897-y)
Supplement: Supplementary file 2 — Additional file 2: Table S2. siRNA sequences. [file 12967_2023_3897_MOESM2_ESM.docx]

**Table S2 | siRNA sequences**

| **Name** | **RNA Oligo Sequences (5' - 3')** |
| --- | --- |
| siCCL3-1 | CCGGCAGATTCCACAGAATTT |
| siCCL3-2 | CGGTGTCATCTTCCTAACCAA |
| siVIRMA-1 | GTATCATCTTCTCTTAAGT |
| siVIRMA-2 | GGACATGCGTGTTCCTTCA |
| siSIRT1-1 | GGAAAUAUAUCCUGGACAATT |
| siSIRT1-2 | GCAACUAUACCCAGAACAUTT |
