# Supplementary material for: CCL3 secreted by hepatocytes promotes the metastasis of intrahepatic cholangiocarcinoma by VIRMA-mediated N6-methyladenosine (m6A) modification
Source: J Transl Med. 2023 Jan 23;21:43. doi: 10.1186/s12967-023-03897-y (PMC9869516; doi:10.1186/s12967-023-03897-y)
Supplement: Supplementary file 3 — Additional file 3: Figure S1. Functional enrichment analysis of cytokine antibody array. Figure S2. Validation of VIRMA knockdown and overexpression in ICC cells. (a) qRT-PCR (up) and western blot (down) analysis of VIRMA expression in VIRMA-knockdown ICC cells. siVIRMA-1 and siVIRMA-2 indicated VIRMA knockdown in both RBE and HuCCT1 cells. (b) qRT-PCR (up) and western blot (down) analysis of VIRMA expression in VIRMA-overexpression ICC cells. oeVIRMA indicated RBE cells and HuCCT1 cells transfected with an overexpressing plasmid. The relative quantification was calculated using the 2−ΔΔCt method and normalized based on GAPDH. Figure S3. MeRIP-seq and RNA-seq analysis. (a) GO analysis of down-regulated genes identified for MeRIP-seq. (b) Motif analysis. (c) The peak annotation for m6A signal enrichment in MeRIP-seq. (d) GO analysis of down-regulated genes identified for RNA-seq. (e) KEGG analysis of down-regulated genes identified for RNA-seq. (f) GO analysis of up-regulated genes identified for RNA-seq. (g) KEGG analysis of up-regulated genes identified for RNA-seq. Figure S4. Quantification of rescue experiments. [file 12967_2023_3897_MOESM3_ESM.docx]

**Figure S1. Functional enrichment analysis of cytokine antibody array.**


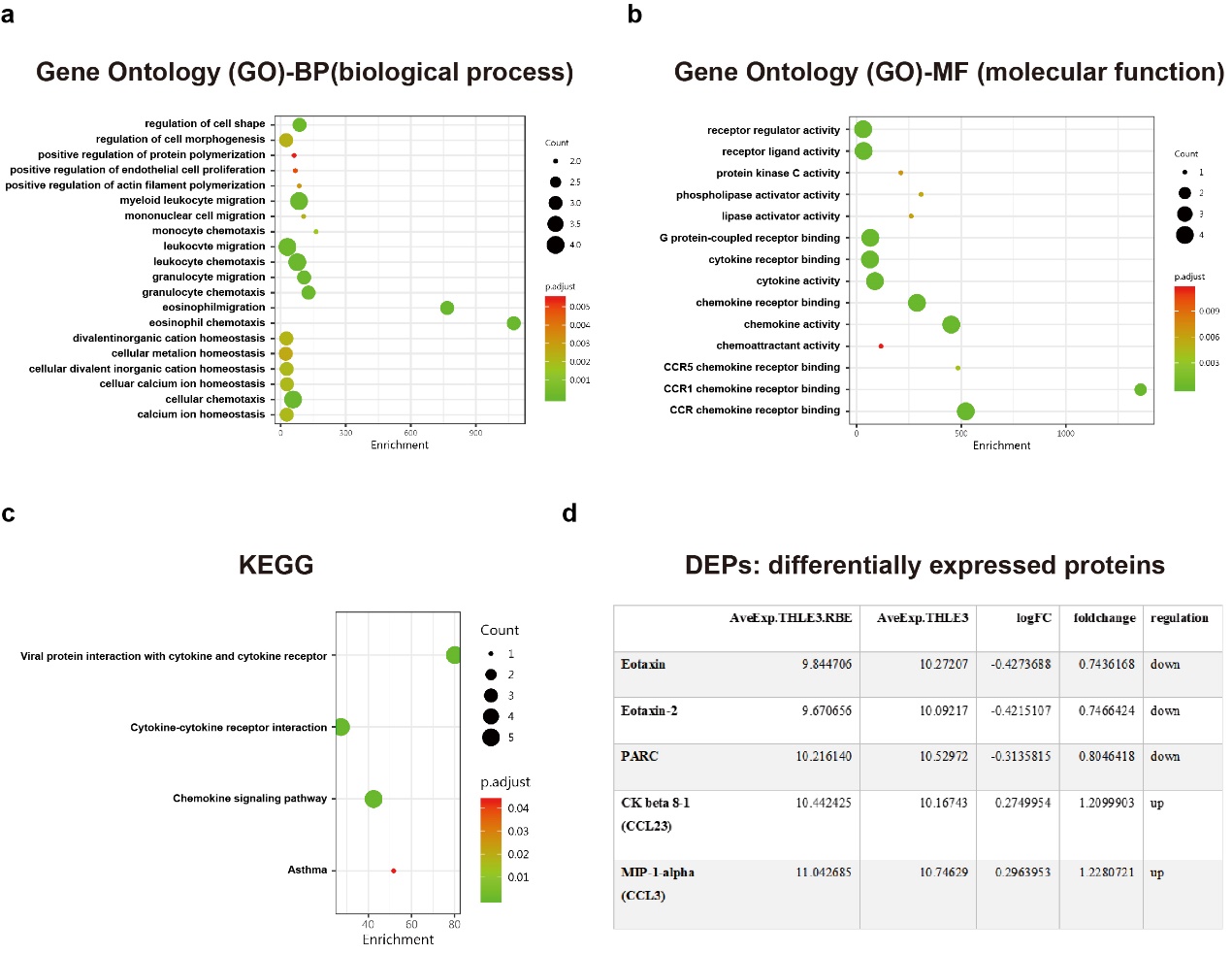


**Figure S2. Validation of VIRMA knockdown and overexpression in ICC cells. (a)** qRT-PCR (up) and western blot (down) analysis of VIRMA expression in VIRMA-knockdown ICC cells. siVIRMA-1 and siVIRMA-2 indicated VIRMA knockdown in both RBE and HuCCT1 cells. **(b)** qRT-PCR (up) and western blot (down) analysis of VIRMA expression in VIRMA-overexpression ICC cells. oeVIRMA indicated RBE cells and HuCCT1 cells transfected with an overexpressing plasmid. The relative quantification was calculated using the 2^−ΔΔCt^ method and normalized based on GAPDH.

**
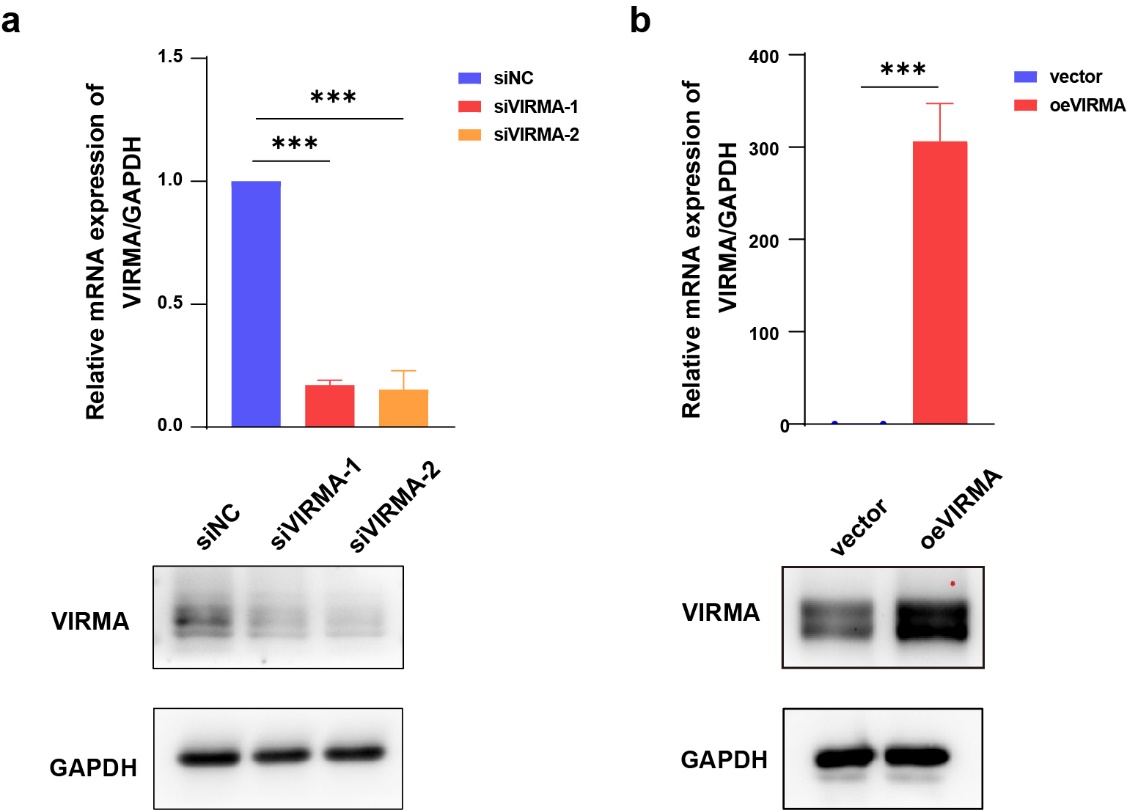
**

**Figure S3. MeRIP-seq and RNA-seq analysis.** **(a)** GO analysis of down-regulated genes identified for MeRIP-seq. **(b)** Motif analysis. **(c)** The peak annotation for m^6^A signal enrichment in MeRIP-seq. **(d)** GO analysis of down-regulated genes identified for RNA-seq. **(e)** KEGG analysis of down-regulated genes identified for RNA-seq. **(f)** GO analysis of up-regulated genes identified for RNA-seq. **(g)** KEGG analysis of up-regulated genes identified for RNA-seq.

**
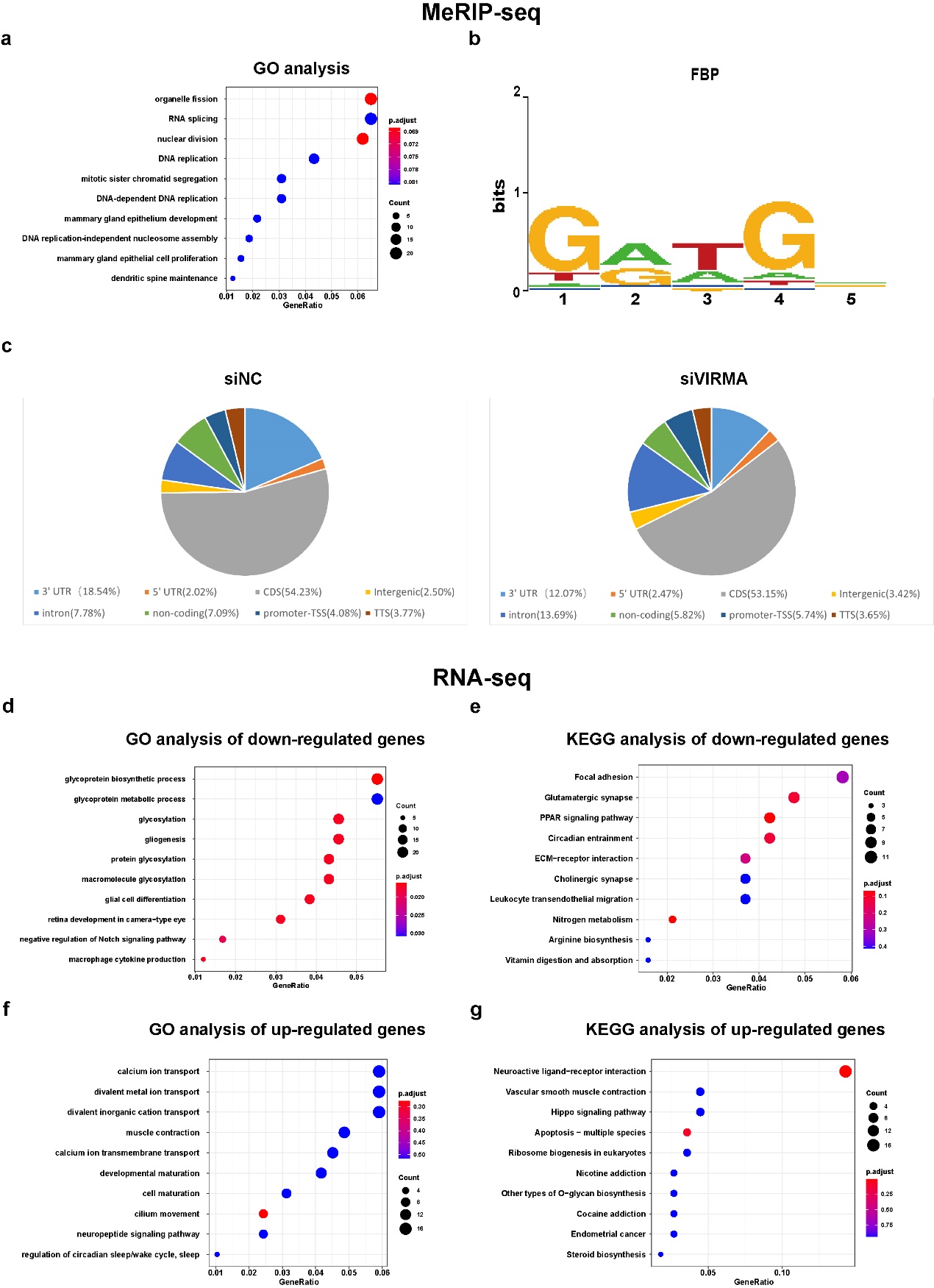
**

**Figure S4. Quantification of rescue experiments.**

**
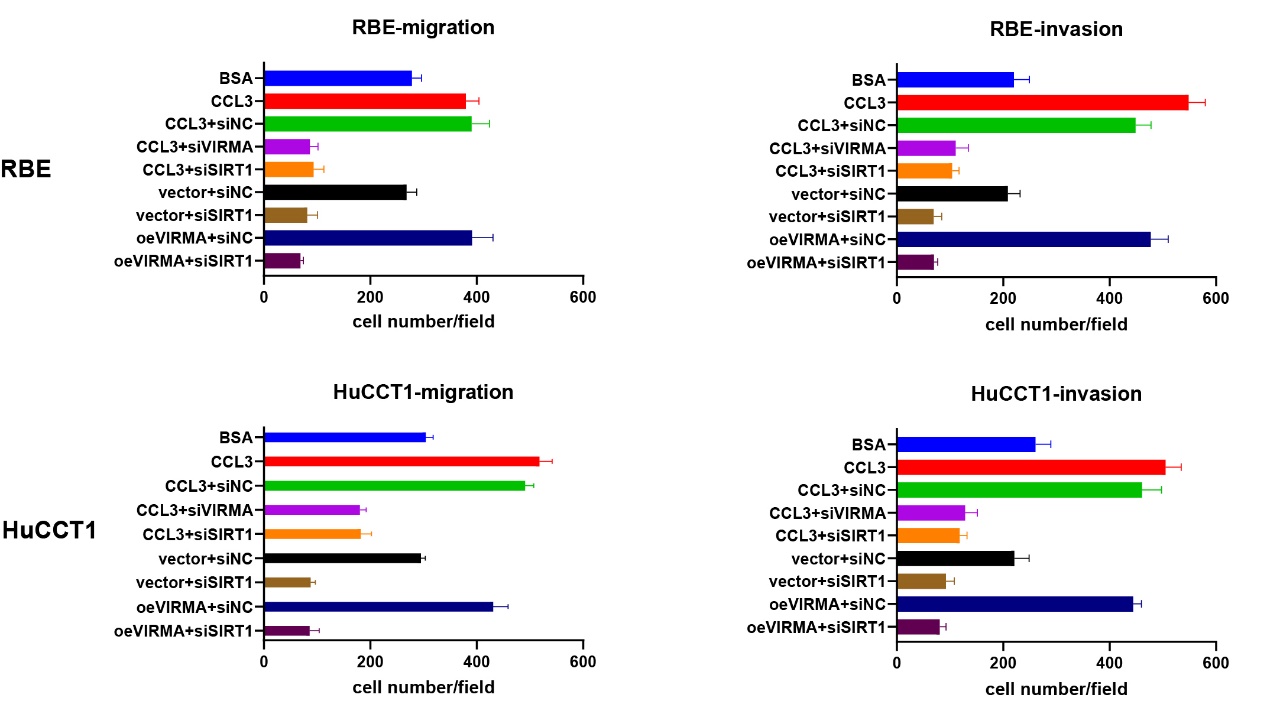
**
