# Supplementary material for: CCL3 secreted by hepatocytes promotes the metastasis of intrahepatic cholangiocarcinoma by VIRMA-mediated N6-methyladenosine (m6A) modification
Source: J Transl Med. 2023 Jan 23;21:43. doi: 10.1186/s12967-023-03897-y (PMC9869516; doi:10.1186/s12967-023-03897-y)
Supplement: Supplementary file 4 — Additional file 4: Additional methods and experimental details. [file 12967_2023_3897_MOESM4_ESM.docx]

**Cell counting kit-8 (CCK-8) assays**

CCK8 assays were performed to evaluate cell proliferation in vitro using the CCK8 Kit (Beyotime Biotechnology, China) on the basis of the manufacturer’s manual. In brief, cells were seeded in 96-well plates with 2 × 10^3^ cells per well. Then 10μl CCK8 solution was added to each well for incubation at 37 °C for 2 h. Finally, the absorbance was measured at 450 nm by a microplate reader.

**Western blotting (WB)**

After extraction of total protein by using RIPA lysate (Solarbio, China), the BCA Protein Assay Kit (Thermo Fisher Scientific, USA) was utilized to measure the protein concentrations. Equal amounts of proteins (20 μg) were separated on a 10% SDS/PAGE gel and electrotransferred (100 V, 2 h) to nitrocellulose membranes. The membranes were blocked with 5% BSA for 1 h and then were incubated with primary antibody overnight at 4 °C. The corresponding secondary antibodies were applied on the following day. The blots containing target bands were exposed to enhanced chemiluminescence (ECL) reagent (Solarbio, China) on the Exposure meter. The densitometry readings of each band were detected by ImageJ software 1.8.0 in the gray value analysis, and the relative expression was calculated as intensity ratio = target protein gray density/GAPDH gray density.

**Immunohistochemistry (IHC)**

Tissue samples from ICC patients as previously mentioned were fixed in formalin, embedded in paraffin, and cut into 4‐μm sections. The paraffin-embedded sections were deparaffinized, hydrated, and blocked. And then the sections were incubated with primary antibodies overnight, followed by secondary antibodies for 2h. Finally, DAB+ chromogen was used for color development and hematoxylin was applied to counterstain. The IHC score for each case was calculated by the following formula: IHC score = positive percentage score × intensity score. The staining percentage score was graded as follows: 0; 0-25% of positively staining cells = 1; 25-50% of positively staining cells = 2; 50-75% of positively staining cells = 3; >75% of positively staining cells = 4. The staining intensity score was graded as follows: negative = 0; low positive = 1; positive = 2; high positive = 3.

**RNA pull-down assay**

RNA was first transcribed by the MEGAscript T7 Transcription Kit (AM1334, Thermo Scientific). The amplified RNA was then end-labeled with desthiobiotin using Pierce RNA 3′End Desthiobiotinylation Kit (20, 163, Thermo Scientific), following the manufacturer’s instructions. RNA pull-down assays were conducted using the Pierce Magnetic RNA-Protein Pull-Down Kit (20164, Thermo Scientific). Up to 50 pmol of biotinylated RNAs were mixed with 2 mg of protein lysate and 50 μl of streptavidin beads. The mixture was incubated and washed three times, and then the streptavidin beads were boiled and used for the immunoblotting assay.

**RNA immunoprecipitation (RIP) assay**

RIP was performed using the Magna RIP RNA-Binding Protein Immunoprecipitation Kit (17–700, Millipore), following the manufacturer’s instructions. Briefly, magnetic beads coated with 5 μg of specific antibodies against mouse immunoglobulin G (17–700, Millipore), or VIRMA (25712-1-AP, Proteintech) were incubated with prepared cell lysates at 4°C overnight. Thereafter, the RNA-protein complexes were washed 6 times and incubated with proteinase K. Finally, RNA was extracted by the phenol-chloroform RNA extraction method. The relative interaction between VIRMA and SIRT1 transcripts was determined by qPCR.

**Statistical analysis**

The study results were presented as a mean ± SD, from at least three independent experiments. Statistical analysis was conducted with SPSS 22.0 software (SPSS, Chicago, Ⅱ, USA) and GraphPad Prism Version 9 (GraphPad, Software, Inc., La Jolla, USA). The Student’s t-test was used to compare differences between two independent groups. Overall survival (OS) and disease-free survival (DFS) analysis were assessed by the Kaplan-Meier method and compared by the log-rank test. A *P* value < 0.05 was considered to indicate a statistically significant difference.
